# Unveiling the Nanoconfinement Effect in CO_2_ Electroreduction to CH_4_ over Mesoporous Cu-CeO_2_ Nanospheres

**DOI:** 10.1021/acscentsci.5c01035

**Published:** 2025-08-22

**Authors:** Lei Xiong, Xianbiao Fu, Wenpu Fan, Jun Zhang, Zixuan Zheng, Shaojie Lu, Dong Wang, Mingze Hao, Qin Yue

**Affiliations:** † Institute of Fundamental and Frontier Sciences, 12599 University of Electronic Science and Technology of China, Chengdu 610054, China; ‡ Department of Materials Science and Engineering, 37580 National University of Singapore, Singapore, Singapore 117576, Singapore; § State Key Laboratory of Green Chemical Engineering and Industrial Catalysis, Centre for Computational Chemistry and Research Institute of Industrial Catalysis, School of Chemistry and Molecular Engineering, 47860 East China University of Science and Technology, 130 Meilong Road, Shanghai 200237, China; ∥ State Key Laboratory of Industrial Vent Gas Reuse, Southwest Institute of Chemical Co., Ltd., Chengdu 610225, China; ⊥ College of Chemistry, 12530 Sichuan University, Chengdu 610040, China

## Abstract

Nanoconfinement provides a promising strategy to promote the electrochemical CO_2_ reduction reaction (CO_2_RR) owing to enhanced reactant enrichment and collision. However, the nanoconfinement influence on the CH_4_ selectivity from the CO_2_RR with related regulation mechanism is unclear. Herein, a series of mesoporous CeO_2_ loaded Cu catalysts with controllable pore size (1.3–5.5 nm) are designed to modulate the CO_2_RR selectivity to CH_4_. It is found that decreasing the pore size can apparently enhance the CO_2_RR performance while inhibiting the HER activity. Moreover, a volcano-type relationship between the CH_4_ selectivity and the pore diameter is observed among these catalysts, while Cu-mCeO_2_-3.0 (pore diameter of 3.0 nm) shows the highest CH_4_ Faradaic efficiency (66.1 ± 2.9%). The in situ experiments and DFT calculations illustrate that a smaller pore size with stronger confinement over Cu-mCeO_2_-*x* can promote the adsorption and transformation of reactants (*CO, *CHO, etc.) for CH_4_ production, but too narrow confined space (1.3 nm) will contribute to much higher intermediate coverage and promote their collision for C–C coupling to C_2+_ products instead, thus reducing the CH_4_ selectivity. This work provides designing insights into metal/oxide catalysts with controllable pore size to study the nanoconfinement effect on the CO_2_RR-to-CH_4_ activity, which can be extended to other oxide-based catalytic reactions.

## Graphical abstract

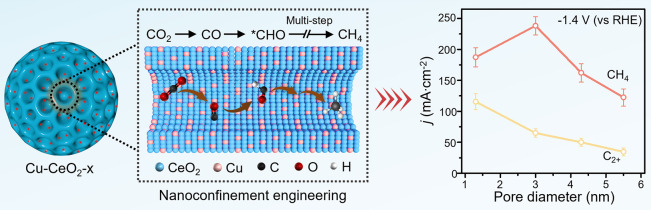

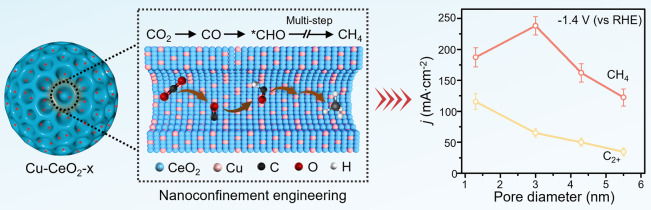

## Introduction

Electrochemical CO_2_ reduction into value-added fuels and chemical feedstocks via renewable electricity (solar and wind) is an appealing technology to lower CO_2_ emission and close the carbon loop.^1–3^ Through multistep coupling of proton and electron transfer, various products have been reported including carbon monoxide (CO),^4^ formate (HCOO^–^),^5^ methane (CH_4_),^6^ ethylene (C_2_H_4_),^7^ and other hydrocarbons (ethanol, acetate, etc.)^8^ over various catalysts. Among them, CH_4_ possesses the highest calorific value (55.5 MJ·kg^–1^), which serves as the key component of natural gas for clean combustion.^9^ Natural gas provides 24% of global energy, electrochemical CO_2_RR to CH_4_ will offer a candidate pathway in the infrastructure of CH_4_ storage, transportation and consumption established worldwide.^10,11^

Up to now, the Cu-based materials have mainly been reported to be capable of catalyzing electrochemical CO_2_ reduction to CH_4_, ascribed to their unique characteristic of moderate binding energy of the key intermediate *CO.^12^ However, the production of CH_4_ with high activity and selectivity is challenging owing to the sluggish process of eight-proton-coupled electron transfer.^13^ Numerous strategies have been proposed to enhance the Faradaic efficiency (FE) and current density of CH_4_ over Cu-based catalysts, consisting of particle-size modulation,^14,15^ oxidation-state manipulation,^16,17^ design of single-atom catalysts,^18,19^ and Cu/oxide interface engineering.^20,21^ The intimate metal/oxide interfacial interactions are suggested to be dominant in significantly improving the molecular adsorption and activation in multiphase catalytic reactions containing the CO_2_RR.^20,22^ Among them, the Cu/CeO_2_ composites have been verified effective to catalyze the CO_2_RR to deep-reduction products (>2e^–^) like C_2+_ (C_2_H_4_, C_2_H_5_OH, etc.) and CH_4_, while the Cu-CeO_2_ solid solution is recognized as the active site for CH_4_ production.^17,23^ However, the competition of the undesirable hydrogen evolution reaction (HER) restricts the CH_4_ selectivity.

Recently, nanoconfinement has been regarded as a viable strategy to enhance the electrocatalytic activity.^24,25^ The term “nanoconfinement” can be defined as confined regions with a reaction environment different from that at the macroscale surface, which can influence the reactant concentration, reactant adsorption energies, and electrolyte composition.^26–28^ As a reaction involving a gas–solid–liquid triphase interface, the CO_2_RR has been verified to be highly affected by the nanoconfinement. For example, Hall et al.^29^ reported that the porous Au electrode could significantly inhibit the HER while preserving high rates of CO_2_RR to CO. After that, Yang et al.^30^ illustrated that the multihollow Cu_2_O could confine carbon intermediates (like *CO) formed in situ in the nanocavities, which promoted the Cu^+^ stabilization and C–C coupling to enhance the C_2+_ selectivity. It is speculated that the nanoconfinement effect may alter the CO_2_ reduction reaction path and intermediate adsorption energy. How the nanoconfinement effect influences the CO_2_RR-to-CH_4_ selectivity, their structure–activity relationship, and regulation mechanism is still unclear; however, it is of important significance.

Herein, this pioneering work investigates the relationship between the nanoconfinement effect and CH_4_ selectivity as well as unravels their regulation mechanism through synthesizing a series of mesoporous CeO_2_ loaded Cu nanospheres with various pore sizes (Cu-mCeO_2_-*x*, *x* = 1.3, 3.0, 4.3, and 5.5 representing the pore size) as the CO_2_RR electrocatalysts. The nanoconfinement effect was influenced by the degree of freedom of which the species entered and left the nanoconfined space, while a lower degree of freedom indicates a greater confinement.^25^ The successful controllable pore size for Cu-mCeO_2_ allows modulation of the nanoconfinement effect, thus further promoting the CO_2_RR performance toward CH_4_ production. The results illustrate that the CO_2_RR performance is improved but HER is inhibited with the decrease of pore size, owing to the enhanced nanoconfinement effect. Moreover, the CH_4_ selectivity shows a volcano relationship with the pore diameter, while Cu-mCeO_2_-3.0 shows the best CH_4_ selectivity with maximum FE_CH4_ of 66.1 ± 2.9% and relative *j*_CH4_ of 237.6 ± 14.5 mA·cm^–2^ at −1.4 V (vs RHE). The in situ spectral and DFT results illustrate that stronger nanoconfinement with small pore size is conducive for the transformation and adsorption of the reactants and intermediates (like CO and *CHO) critical for CH_4_ production, but too high intermediate coverage within a very narrow pore space (1.3 nm) will conversely promote the intermediate collision for C–C coupling to produce C_2+_, thus decreasing the CH_4_ Faradaic efficiency instead.

## Results and Discussion

Mesoporous CeO_2_ spheres loaded on Cu with independent mesopores (Cu-mCeO_2_-*x*, *x* = 1.3, 3.0, 4.3, and 5.5 nm representing the mesopore size) were synthesized to achieve highly efficient CO_2_RR toward CH_4_ production via a molten salt assisted assembly strategy followed by the impregnation method (Figure 1a). The solid mixtures of Ce­(SO_4_)_2_, block copolymer F127, and nitrate (KNO_3_ and NaNO_3_) via grinding are calcined in a muffle furnace under an air atmosphere.^31^ The temperature is first raised to 160 °C and maintained there for 3 h, while the mixed nitrate (molar ratio KNO_3_:LiNO_3_ = 0.57:0.43) melts into a stable liquid to serve as the synthesis medium owing to its low eutectic temperature (∼125 °C) and superior stability.^32^ The surfactant F127 will coordinate with Ce^4+^ via its ether oxygen and aggregate into micelles in the hydrophilic molten salt, thus generating mesostructured organic–inorganic hybrid complexes. After further increasing the temperature to 400 °C, the crystallized CeO_2_ forms accompanied by the complete decomposition of F127, as verified by the TG-DTG curves (Figure S1), obtaining the mesoporous structure. The molten salt formed at high temperature can provide a stable liquid-phase environment to inhibit the growth of CeO_2_ crystals into large particles, thus generating highly crystallized mesoporous CeO_2_. Finally, the Cu-mCeO_2_ catalyst is prepared via a simple impregnation method and annealing treatment at 400 °C.

**Figure 1:**
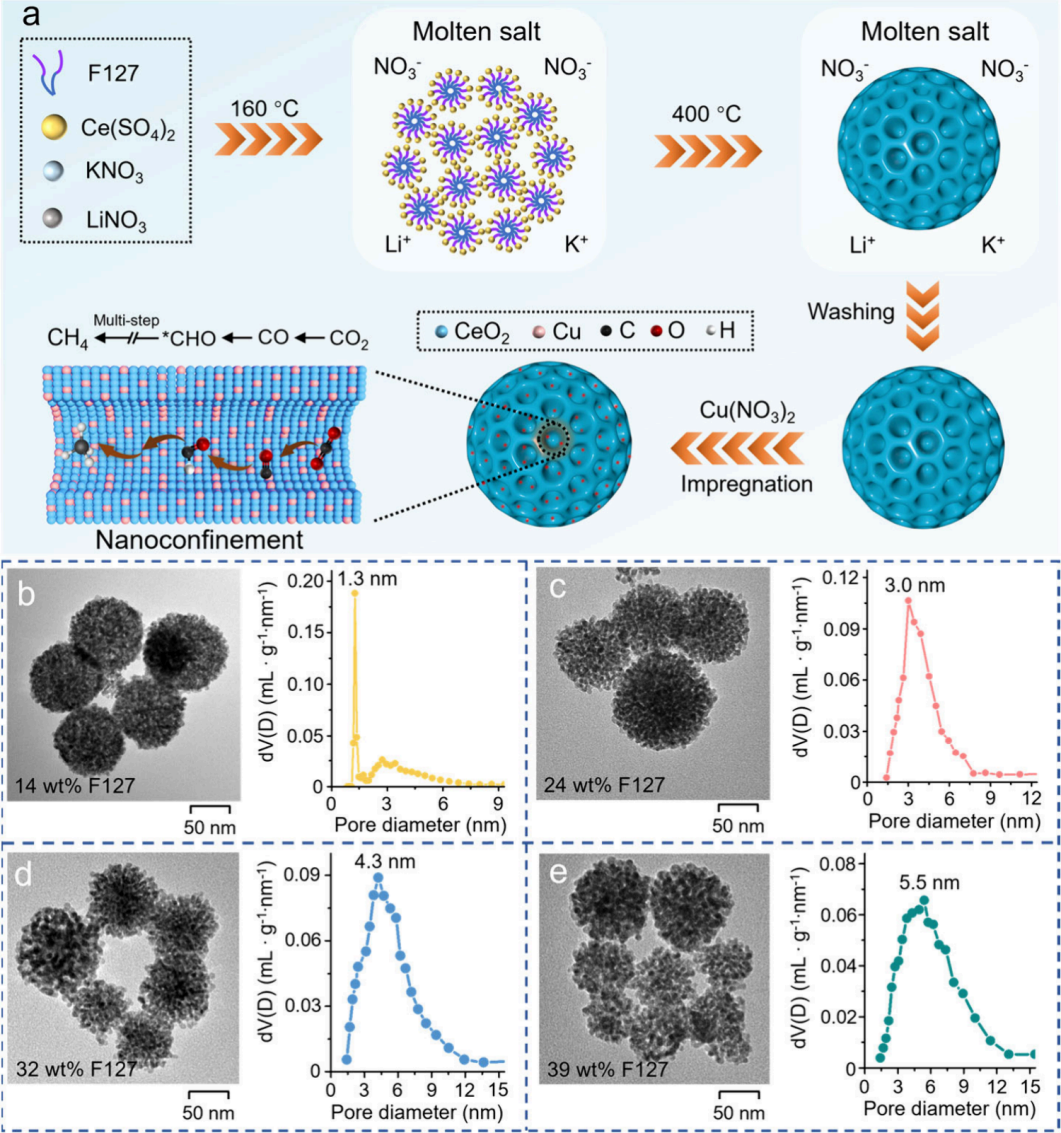
(a) Schematic diagram of synthesis procedures for the porous Cu-CeO_2_ as a nanoreactor applied to the CO_2_RR. TEM images and relative pore size distributions of Cu-mCeO_2_-1.3 (b), Cu-mCeO_2_-3.0 (c), Cu-mCeO_2_-4.3 (d), and Cu-mCeO_2_-5.5 (e).

The TEM (Figure 1b–e) and SEM (Figure S2) images reveal the porous nanospheres for all of the Cu-mCeO_2_ catalysts. Moreover, with the increase of F127 amount, the pores become significantly larger centered at 1.3, 3.0, 4.3, and 5.5 nm, as confirmed by the pore size distribution obtained from the N_2_ adsorption–desorption isotherm (Figure S3). As a result, the relative catalysts are denoted as Cu-mCeO_2_-*x* (*x* = 1.3, 3.0, 4.3, and 5.5 nm). The BET surface areas are determined to be 165, 151, 140, and 126 m^2^·g^–1^ for Cu-mCeO_2_-1.3, Cu-mCeO_2_-3.0, Cu-mCeO_2_-4.3, and Cu-mCeO_2_-5.5, respectively (Table S1). These results verify the controllable regulation of the pore size for the Cu/mCeO_2_-*x* catalysts, which can serve as a new model to investigate the nanoconfinement effect over the CO_2_RR activity and product selectivity.

From the HRTEM and relative SAED images (Figure 2a and Figure S4), only the lattice spacing of about 0.315 nm and diffraction rings corresponding to the cubic CeO_2_ are observed for all the Cu-CeO_2_-*x* catalysts. Moreover, the EDS mappings (Figure 2b and Figure S5) validate the relatively uniform Cu dispersion in the pores of CeO_2_. The actual Cu contents are determined to be 3.0, 3.1, 3.2, and 3.0 wt % for Cu-mCeO_2_-1.3, Cu-mCeO_2_-3.0, Cu-mCeO_2_-4.3, and Cu-mCeO_2_-5.5 via ICP-OES, respectively, which are close to their theoretical values. The powder X-ray diffraction (XRD) patterns (Figure 2c) of all the Cu-CeO_2_-*x* catalysts display only the diffraction peaks ascribed to the fluorite-like cubic CeO_2_ (PDF#34-0394), verifying the high dispersion of Cu. Raman spectra were also employed to characterize the structure of Cu-CeO_2_-*x* as shown in Figure 2d. A sharp peak at ∼452 cm^–1^ and a less intense band at 593 cm^*‑*1^ are observed for all the Cu-CeO_2_-*x* species, ascribed to the F_2g_ mode and Frenkel defects in the CeO_2_ fluorite lattice, respectively, illustrating the formation of Cu-CeO_2_ solid solution.^17,21^ In situ CO diffuse reflectance infrared Fourier transform spectroscopy (CO Drifts) was employed to investigate the physical state of Cu (Figure S6). Mainly two peaks located at ∼2175 cm^–1^ and ∼2063 cm^–1^ are observed for all four Cu-CeO_2_-*x* catalysts, ascribed to CO adsorbed on CeO_2_ and the atop adsorption over Cu (Cu-CO_atop_), respectively.^33,34^ Nevertheless, no peak appears at 1900–1800 cm^–1^ that belongs to the bridge-bonded CO species.^35,36^ It is well recognized that the Cu-CO_atop_ corresponds to linear adsorption over Cu single sites, while Cu-CO_bridge_ is associated with adsorbed CO coordinated to multiple Cu atoms.^34,36^ Therefore, Cu mainly exists as single-atom species for all the Cu-CeO_2_-*x* catalysts.

**Figure 2:**
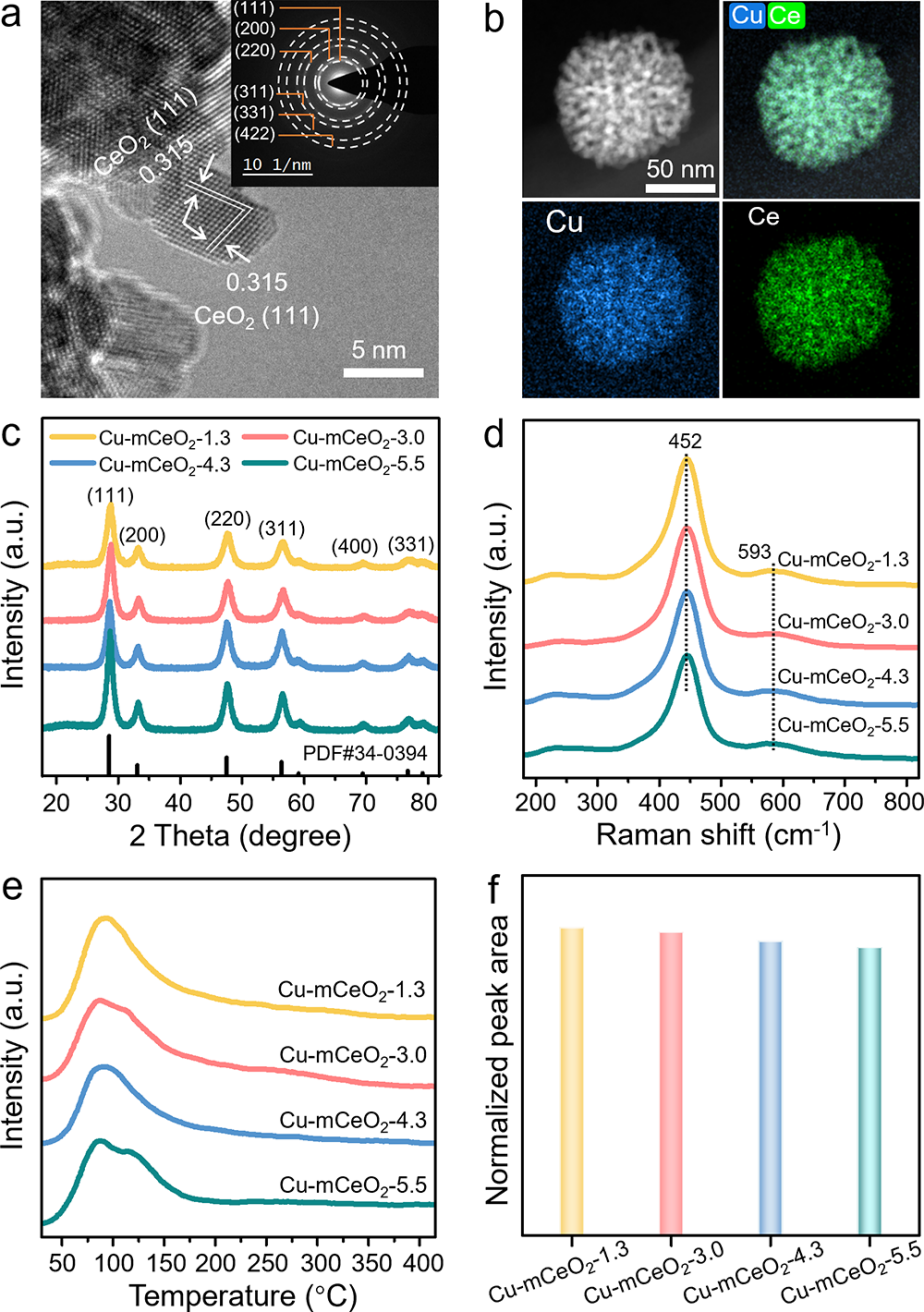
Physiochemical characterizations of the catalysts. The HRTEM image (inset is the SAED) (a) and EDS mapping (b) of Cu-mCeO_2_-3.0. The XRD patterns (c) and Raman (d) of the four catalysts. The CO_2_-TPD spectra (e) and relative BET surface area normalized peak area (f) of Cu-mCeO_2_-*x*.

XPS was used to characterize the electronic structure of elements like Cu, O, and Ce, as shown in Figure S7. The Cu 2p (Figure S7a) and Cu LM2 spectra (Figure S7b) mainly show the coexistence of Cu^+^ and Cu^2+^ among these four catalysts, while Cu^+^ is dominant with a relative content above 80% (Table S2). Moreover, the O 1s XPS spectra are deconvoluted into three peaks (Figure S7c), including lattice oxygen (O_L_) at 529.4 eV, vacancy oxygen (O_v_) at 531.7 eV, and surface oxygen (O_S_) at 533.1 eV, respectively.^37^ The relative O_v_ contents are calculated to be 26.8%, 24.7%, 21.1%, and 19.2% for Cu-mCeO_2_-1.3, Cu-mCeO_2_-3.0, Cu-mCeO_2_-4.3, and Cu-mCeO_2_-5.5 (Table S2), respectively, which are positively correlated with the surface area. CO_2_-TPD was employed to investigate the CO_2_ adsorption ability of the catalysts. As shown in Figure 2e, mainly one broad desorption peak at 25–200 °C is observed for these four catalysts, ascribed to the weak CO_2_ chemisorption.^38^ The relative desorption peak area (Figure S8) is normalized to the BET surface area, which displays little difference among the four Cu/mCeO_2_-*x* catalysts (Figure 2f), illustrating that different surface areas and pore sizes do not influence the intrinsic chemisorption of reactants for these four catalysts with the same Cu loading.

The CO_2_RR performance of the Cu-CeO_2_-*x* catalysts was tested in a homemade flow cell with 0.5 M KHCO_3_ as electrolyte via the chronoamperometry method with bias from −0.6 V to −1.6 V (vs RHE). As shown in Figure 3a–d, the products detected consist of H_2_, CO, HCOOH, CH_4_, and C_2+_ (C_2_H_4_, EtOH, and acetate), while CH_4_ is dominant among all of the catalysts. Moreover, with the decrease of pore diameter, the CO_2_RR (Figure S9) and HER performances (Figure S10) display an increasing and decreasing tendency, respectively. Specifically, the CH_4_ (Figure 3e) and C_2+_ selectivities (Figure S11) display volcano-type and increasing tendency with the decreasing pore size, respectively, while Cu-mCeO_2_-3.0 shows the best CH_4_ selectivity (Max FE_CH4_ of 66.1 ± 2.9% with *j*_CH4_ of 237.6 ± 14.5 mA·cm^–2^ at −1.4 V (vs RHE)). To confirm the role of pore nanoconfinement in the CO_2_RR activity, we further compare the commercial nonporous CeO_2_ loaded Cu with the same content of 3 wt % (Cu-cCeO_2_) (Figure S12). Nearly no apparent pore structure is observed for Cu-cCeO_2_ from the TEM image (Figure S12b). Moreover, Cu-cCeO_2_ shows a much lower current density of *j*_total_ and *j*_CH4_ than the other four Cu-mCeO_2_-*x* catalysts, owing to its much higher HER competition (Figure S12c,d). It verifies that the nanoconfinement of the mesopore structure can indeed contribute to the enhanced CO_2_RR activity for Cu-mCeO_2_-*x* catalysts. For the CO_2_RR performance of the pristine CeO_2_ (Figure S13), mainly H_2_ and CO are detected, while H_2_ is the dominant product. That means Cu is the only active site for the CO_2_RR to generate value-added products.

**Figure 3:**
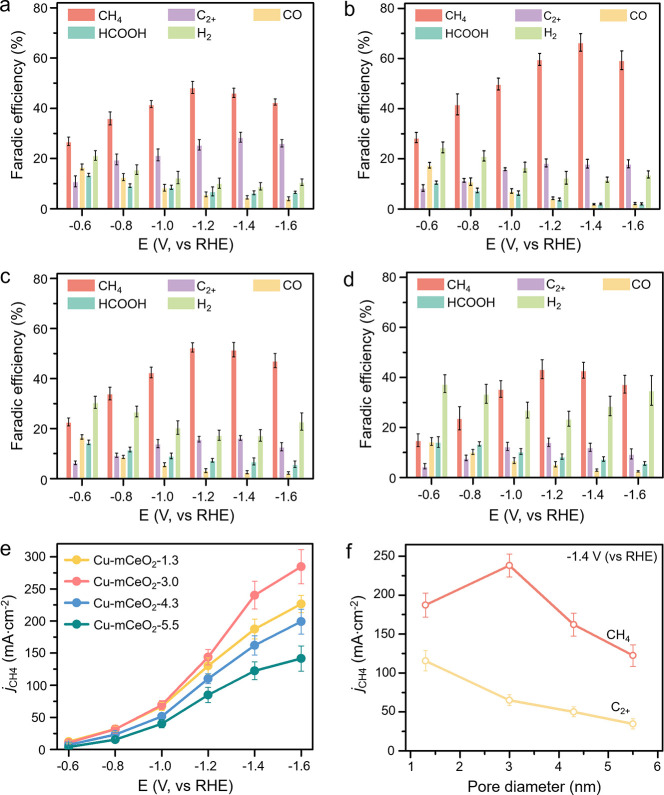
CO_2_RR performance of the catalysts. FEs for (a) Cu-mCeO_2_-1.3, (b) Cu-mCeO_2_-3.0, (c) Cu-mCeO_2_-4.3, and (d) Cu-mCeO_2_-5.5. (e) Relative *j*_CH4_ values of the four catalysts. (f) Correlation between the pore size and *j*_CH4_ and *j*_C2+_ at −1.4 V (vs RHE).

The nanoconfinement refers to the increased collisions and retention due to short diffusion lengths, steric hindrance, and reactant accumulation, so the factor of the electrochemical active surface area (ECSA) should be excluded to study the influence of the pore confinement over the intrinsic activity. The Pb underpotential deposition (UPD) method is employed to estimate the ECSA of Cu-based catalysts. As shown in Figure S14, the area of a Pb monolayer stripping peak around −0.3 V vs Ag/AgCl was integrated to calculate the transferred charge. The calculated ECSAs are 0.92, 0.89, 0.85, and 0.82 cm^2^ for Cu-mCeO_2_-1.3, Cu-mCeO_2_-3.0, Cu-mCeO_2_-4.3, and Cu-mCeO_2_-5.5, respectively. The closed ECSA values among these four catalysts indicate that the interfacial Cu/CeO_2_ sites are actually electrochemically active, which are closely related to the dispersion and loading content of Cu. After ECSA normalization, it is noted that the *j*_CH4_ (Figure S15) and *j*_C2+_ (Figure S16) still display a volcano and linear relationship with the pore size, respectively, where Cu-mCeO_2_-3.0 shows the best CH_4_ selectivity.

Electrochemical impedance spectra (EIS) (Figure S17) of Cu-CeO_2_-*x* catalysts were measured under −0.8 V (vs RHE). The charge transfer resistance (R_ct_) follows the order: Cu-CeO_2_-1.3 *<* Cu-CeO_2_-3.0 *<* Cu-CeO_2_-4.3 *<* Cu-CeO_2_-5.5 (Table S3), indicating that smaller pore size is conducive for the charge transfer. The long-term stability (Figure S18) of Cu-CeO_2_-3.0 was tested in a MEA device under a current density of 200 mA·cm^–2^. The FE_CH4_ still remains above 50% after 32 h of electrolysis, exhibiting excellent stability under high current densities. After stability tests, morphological and structural characterizations including Raman, XPS, and EDS mappings were conducted. For the Raman spectra (Figure S19), the sharp peak at ∼452 cm^–1^ with a less intense band at 593 cm^*–*1^ verifies the stability of the Cu-CeO_2_ solid solution. The Cu 2p (Figure S20a) and Cu LM2 spectra (Figure S20b) mainly show the coexistence of Cu^+^ and Cu^2+^ among these four catalysts, while Cu^+^ is still dominant with relative contents of 81.7%, 82.8%, 82.9%, and 84.2% (Table S4) for Cu-mCeO_2_-1.3, Cu-mCeO_2_-3.0, Cu-mCeO_2_-4.3, and Cu-mCeO_2_-5.5, respectively, illustrating the stable valence state after CO_2_RR. The relative Cu contents are 2.96, 3.06, 3.02, and 2.91 wt % for Cu-mCeO_2_-1.3, Cu-mCeO_2_-3.0, Cu-mCeO_2_-4.3, and Cu-mCeO_2_-5.5, respectively. Moreover, the EDS mappings (Figure S21) validate the relatively uniform Cu dispersion in the pores of CeO_2_ for all of the Cu-mCeO_2_-*x* catalysts. The above results illustrate that the Cu-mCeO_2_-*x* catalysts are stable under CO_2_RR conditions.

We further extended the study of pore modulation on ECR performance to the Cu-ZrO_2_ composites. Similar to the synthesis of Cu-CeO_2_, three Cu-mZrO_2_-*x* catalysts with a Cu loading content of 3 wt % were prepared, assisted with various F127 contents. The pore diameter centers at 1.2, 2.8, and 4.0 nm (Figure S22) are labeled as Cu-mZrO_2_-1.2, Cu-mZrO_2_-2.8, and Cu-mZrO_2_-4.0, while the related Cu amounts are determined to be 2.92, 3.0, and 2.97 wt % via the ICP-OES measurements, respectively. The CO_2_RR performance of the Cu-ZrO_2_-*x* catalysts was also tested in a homemade flow cell with 0.5 M KHCO_3_ as electrolyte via a chronoamperometry method with bias from −0.6 V to −1.4 V (vs RHE). One can see that the CO_2_RR performance also displays an increasing tendency with the decline of pore diameter (Figure S23), while CH_4_ and H_2_ are the dominant products. Moreover, the C_2+_ and CH_4_ selectivities for Cu-ZrO_2_-*x* catalysts (Figure S24) display increasing and volcano-type tendency with the decreasing pore size, respectively, which are similar to the results of Cu-CeO_2_-*x* catalysts. As a result, Cu-mZrO_2_-2.8 shows the best CH_4_ selectivity with an apparently higher FE_CH4_ of 36.3 ± 2.1% and relative *j*_CH4_ of 72.0 ± 7.5 mA·cm^–2^ at −1.2 V (vs RHE). Therefore, the optimal pore size of ∼3.0 nm is also suitable for the Cu-ZrO_2_ system. Nevertheless, Cu-ZrO_2_ is not an ideal ECR catalyst to yield value-added products owing to its high HER competition activity.

In situ attenuated total reflection-surface-enhanced infrared absorption spectroscopy (ATR-SEIRAS) was employed to study the intrinsic reason for the various CO_2_RR performance over Cu-CeO_2_-1.3, Cu-CeO_2_-3.0, and Cu-CeO_2_-5.5. As shown in Figure 4a–c, four common infrared absorption peaks located at 2190–2110, 1660–1610, 1480–1430, and 1420–1340 cm^–1^ are observed for all the catalysts, which are attributed to the *CO, *H–O–H, *CHO, and *COO^–^ species, respectively.^17,39^ Among them, *COO^–^ is the intermediate for CO_2_ to *CO. The relative peak intensity of COO^–^ follows in the order of Cu-mCeO_2_-1.3 > Cu-mCeO_2_-3.0 > Cu-mCeO_2_-5.5 regardless of the applied bias, illustrating the strong nanoconfinement effect under smaller pores conducive for the CO_2_ activation and transformation.

**Figure 4:**
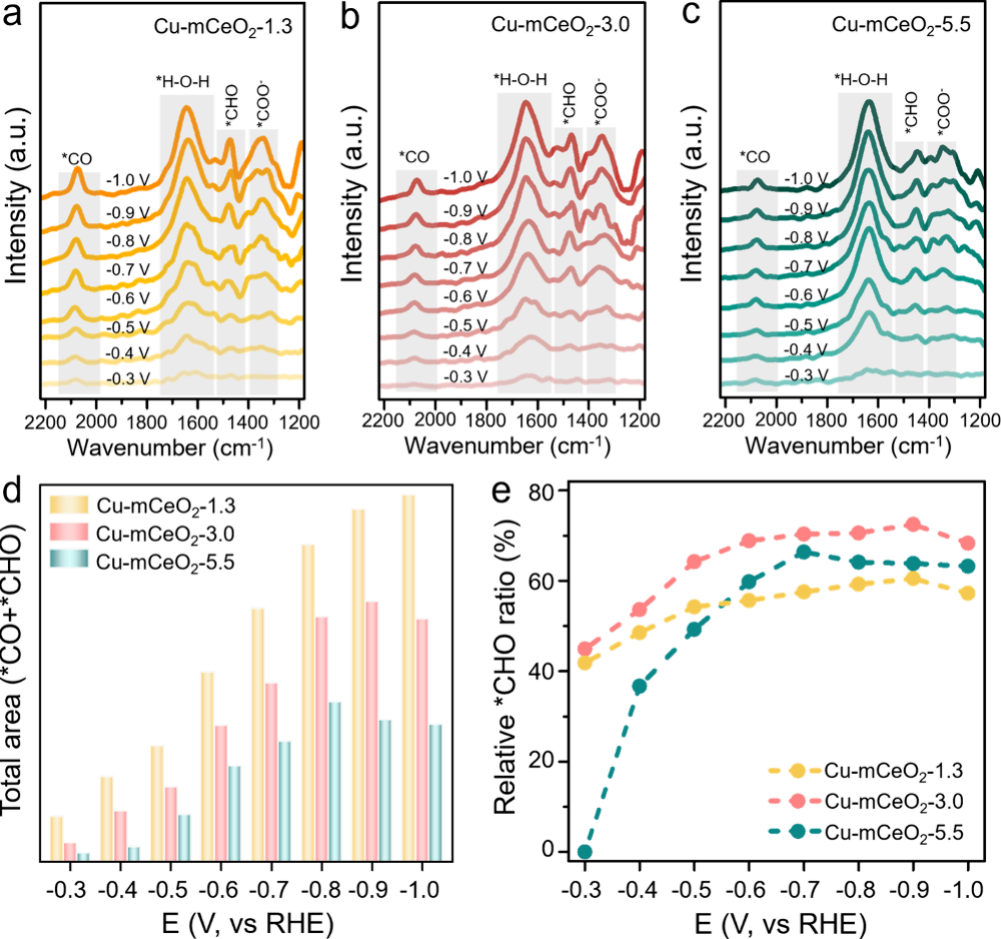
In situ ATR-SEIRAS spectra of Cu-CeO_2_-1.3 (a), Cu-CeO_2_-3.0 (b), and Cu-CeO_2_-5.5 (c). (d) Total peak area of *CO and *CHO. (e) Relative *CHO ratio in the total area of *CO and *CHO.

Moreover, the intermediates *CO and *CHO are recognized as the key intermediates for the CO_2_ deep reduction (>2e^–^), while *CO hydrogenation to *CHO is regarded as the rate-determining step for CH_4_ production.^40–42^ Therefore, the relative peaks of Cu-CeO_2_-1.3, Cu-CeO_2_-3.0, and Cu-CeO_2_-5.5 are further compared as shown in Figure 4d,e. The total peak areas (Figure 4d) of these two intermediates (*CO and *CHO) are gradually increased as the potential becomes more negative over these three catalysts owing to the enhanced reaction rate, while the values are increased with the decrease of pore diameter regardless of potential. This means that a smaller pore size is more conducive for the CO_2_ transformation to *CO and *CHO for further reduction ascribed to the stronger nanoconfinement, verifying the constantly enhanced CO_2_RR performance with decreasing pore diameter (Figure S9). The relative ratios of *CHO intermediates for these catalysts are obtained based on their peak area to the total area of these two intermediates (Figure 4e). With the bias becoming more negative, the *CHO ratio first increases and then remains relatively stable. Furthermore, Cu-CeO_2_-3.0 displays the highest *CHO ratio, indicating fast *CO hydrogenation to *CHO for high CO_2_RR selectivity to CH_4_. However, Cu-CeO_2_-1.3 shows a similar or even smaller *CHO ratio with Cu-CeO_2_-5.5 under high bias even though it has the highest content of these two intermediates, which may be ascribed to the limited active Cu sites and the rate-limiting step (*CO → *CHO) for further *CO hydrogenation. Therefore, high coverage of untimely hydrogenated *CO in the much smaller pores might promote its coupling with *CHO and thus enhance the C_2+_ production instead, accounting for its lower CH_4_ FE but much higher C_2+_ selectivity than that of Cu-CeO_2_-3.0 (Figure 3, Figure S11).

We carried out DFT calculations to capture the confinement effects in those mesoporous materials. To simulate the confinement effect in mesopores of experimental materials, we adopted a representative model engaging one-dimensional confined space in *z* axis, which has been proven effective in capturing intermolecular interactions in confined nanospace.^43–45^ One may notice that the actual confinement perceived by reactants is often more intense than the nominal pore sizes for the more restricted diffusion pathway in the three-dimensional channels with much lower freedom degree.^25^ Thus, in addition to the minimum pore size of 13 Å in experimental materials, we also considered even smaller sizes of 6 and 9 Å as well as larger ones (18 and 24 Å) to account for different degrees of confinement effects in those porous materials (Figure 5a­(2–6); see details in the Experimental Section in the Supporting Information). The adsorption energy of the key intermediates (*H, *CO, *CHO, and *COOH) on the Cu_1_/CeO_2_(111) surface within varying confined spaces (6, 9, 13, 18, and 24 Å) is shown in Figure 5b. Although the adsorption structure generally remains quite similar, the adsorption energy shows evident enhancement (i.e., more negative adsorption energies) in the smallest confined space of 6 Å, whereas the results are comparable among larger spaces for all intermediates. Among them, the adsorption of CO_2_RR intermediates (*COOH, *CO, and CHO) are much superior to that of *H with much negative adsorption energy, confirming the priority of CO_2_RR than HER. These findings support that spatial confinement can effectively stabilize the CO_2_RR intermediates and therefore enhancing their surface coverage under reaction conditions.

**Figure 5:**
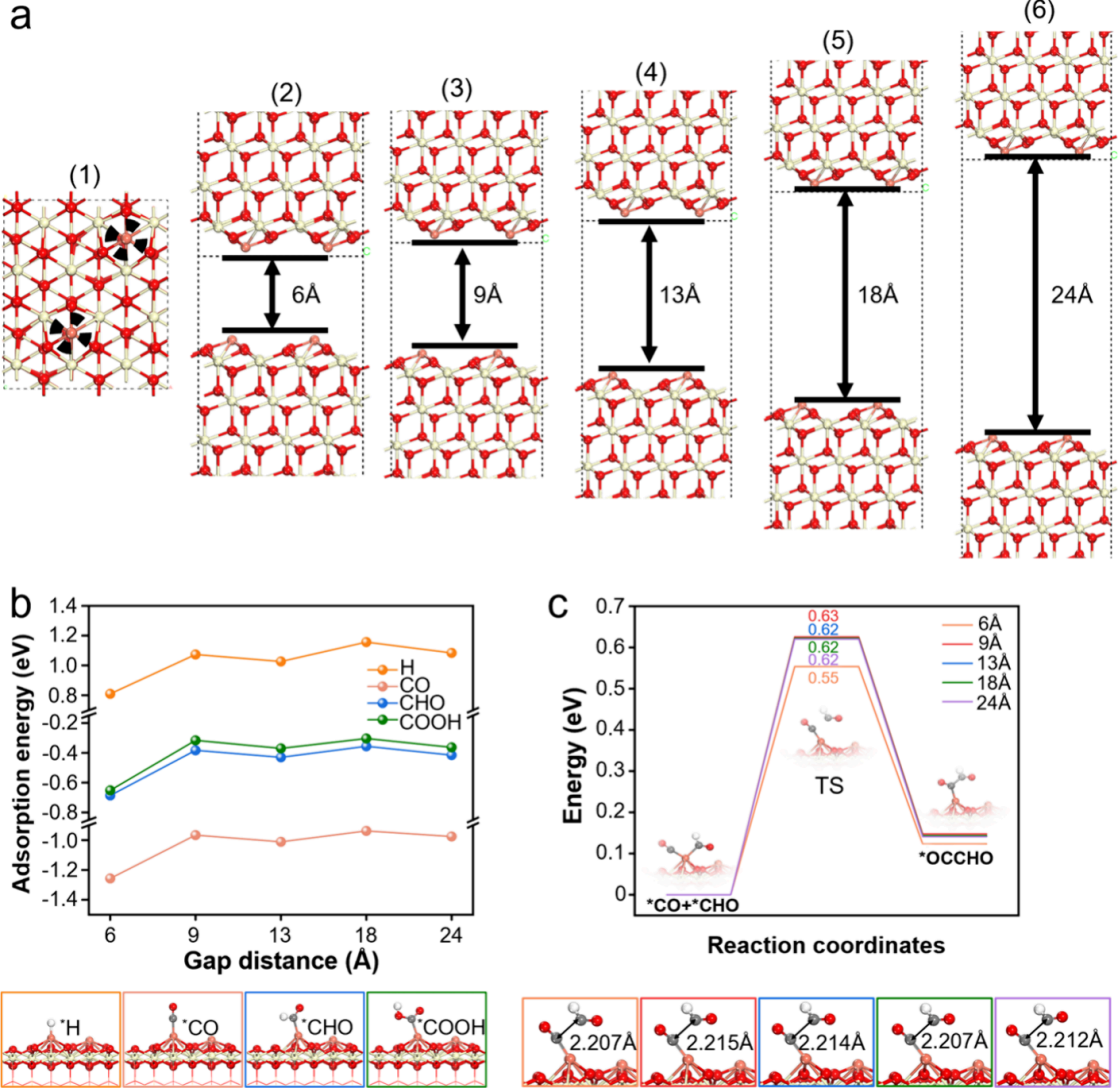
Mechanism study via DFT calculations with different pore diameters. (a) Optimized structures of Cu_1_/CeO_2_(111) with a 3 wt % Cu mass fraction: (1) top view of the catalyst surface and (2)–(6) representative slab models engaging one-dimensional confined space to simulate the confinement effect in cylindrical pores of experimental materials. (b) Calculated adsorption energies and structures of *H, *CO, *CHO, and *COOH, as well as (c) reaction barriers for the coupling of *CO and *CHO to produce C_2+_ within varying confined spaces. Ivory, red, white, gray, and orange balls represent Ce, O, H, C, and Cu atoms, respectively.

Moreover, the reaction energetics of *CO + *CHO → *OCCHO within varying confined spaces were also calculated to evaluate the impact of spatial confinement on the key C–C coupling step (Figure 5c). It is suggested that the reaction begins with *CO and *CHO coadsorption at the same Cu atom and produces the *OCCHO species binding on the surface through the C atom in the −CO group. While the transition state structures in all confinement models exhibit similar geometries with a C–C transition distance of ∼2.2 Å, the determined reaction barrier shows an evident decrease (to be 0.55 eV) in the smallest confined space of 6 Å whereas barriers of 0.62 eV are comparable among larger ones. In addition, we also considered the influence of water environment on reaction energetics of the key *CO/*CHO coupling step, by explicitly introducing local water molecules (the first solvation shell) around the reaction center.^46–50^ Despite a general increase of 0.2–0.3 eV in *CO/*CHO coupling barriers (Figure S25), probably owing to the constraint effect of H-bond network on surface reactants,^51–53^ the general trend of stronger spatial confinement favoring lower barriers remains well maintained under solvated conditions, being 0.72 and 0.87 eV for the 6 and 18 Å confinement models, respectively. Note that although the 6 Å confinement model does not directly correspond to experimental pore diameters, it captures the stronger confinement perceived by reactants at the off-centerline position of cylindrical pores, while the smaller nanopores (like 1.3 nm of the experimental materials) have much narrower off-centerline space. Hence, even though simplified one-dimensional confinement models are used, the calculated CO/CHO coupling energetics show evidently that a more confined space not only stabilizes reaction intermediates (Figure 5b) but also facilitates their interaction and coupling (Figure 5c), leading to improved C_2+_ production. Nevertheless, the DFT calculations can still reflect that a small pore size of the Cu/CeO_2_ is conducive for the CO_2_RR activity, while too narrow a pore width (1.3 nm here) will apparently enhance the C_2+_ selectivity.

Combining the results of in situ ATR-SEIRAS and DFT calculations, it is illustrated that the enhanced nanoconfinement effect by the decreasing pore size can significantly promote CO_2_RR to produce hydrocarbons (CH_4_, C_2+_, etc.) but inhibit the HER side reaction, which is ascribed to the increased adsorption, accumulation, and collisions of CO_2_ and intermediates (like *CO, *CHO) due to the short diffusion lengths.^54^ A smaller nanopore with strong nanoconfinement over Cu-CeO_2_ based composites is conducive for the CH_4_ production due to the promoted CO_2_ transformation to *CO and *CHO intermediates toward the CH_4_ pathway. However, an ultranarrow pore space (mean diameter of 1.3 nm) could contribute to much higher intermediate coverage (*CO and *CHO) and facilitate their interaction and coupling, thus promoting C_2+_ production to a certain extent.

## Conclusion

This work systematically investigated pore-size-dependent nanoconfinement effects in the CO_2_RR-to-CH_4_ reaction using precisely engineered Cu-CeO_2_ catalysts (1.3–5.5 nm pore sizes). Key findings reveal the following (1) A distinct volcano relationship between CH_4_ selectivity and pore diameter is built for the first time. (2) Optimal nanoconfinement in 3.0 nm pores enhances the *CHO/*CO ratio for CH_4_ production (66.1% ± 2.9% FE, 237.6 ± 14.5 mA cm^–2^), while excessive confinement (1.3 nm) promotes C–C coupling to C_2+_. (3) Combined experimental and theoretical evidence demonstrates pore-size-tunable intermediate stabilization as the governing mechanism. These findings establish pore-size engineering as a powerful strategy for regulating the CO_2_RR selectivity in oxide-supported catalysts.

## Supplementary Material



## Notes

Experimental details, TG-DTG curve, SEM/TEM images, in situ CO Drifts, N_2_ adsorption and desorption isotherms, XPS spectra, XRD patterns, Raman spectra, electrochemical performance, and DFT calculations (PDF)

## References

[1] Shin H., Hansen K. U., Jiao F. (2021). Techno-economic assessment of low-temperature
carbon dioxide electrolysis. Nat. Sustain..

[2] Ross M. B., De Luna P., Li Y. F., Dinh C. T., Kim D., Yang P., Sargent E. H. (2019). Designing
materials for electrochemical
carbon dioxide recycling. Nat. Catal..

[3] Birdja Y. Y., Perez-Gallent E., Figueiredo M. C., Gottle A. J., Calle-Vallejo F., Koper M. T. M. (2019). Advances and challenges in understanding the electrocatalytic
conversion of carbon dioxide to fuels. Nat.
Energy..

[4] Xiong L., Fu X., Zhou Y., Nian P., Wang Z., Yue Q. (2023). Precise Site-Hydrophobicity
Modulation for Boosting High-Performance CO_2_ Electroreduction. ACS Catal..

[5] Fu X. B., Wang J. A., Hu X. B., He K., Tu Q., Yue Q., Kang Y. J. (2022). Scalable chemical interface confinement
reduction BioBr
to bismuth porous nanosheets for electroreduction of carbon dioxide
to liquid fuel. Adv. Funct. Mater..

[6] Xu Z., Lu R., Lin Z.-Y., Wu W., Tsai H.-J., Lu Q., Li Y. C., Hung S.-F., Song C., Yu J. C., Wang Z., Wang Y. (2024). Electroreduction
of CO_2_ to methane with triazole molecular catalysts. Nat. Energy..

[7] Garcia
de Arquer F. P., Dinh C.-T., Ozden A., Wicks J., McCallum C., Kirmani A. R., Nam D.-H., Gabardo C., Seifitokaldani A., Wang X., Li Y. C., Li F., Edwards J., Richter L. J., Thorpe S. J., Sinton D., Sargent E. H. (2020). CO_2_ electrolysis to multicarbon products
at activities greater than 1 A cm^–2^. Science.

[8] Luc W., Fu X. B., Shi J. J., Lv J. J., Jouny M., Ko B. H., Xu Y. B., Tu Q., Hu X. B., Wu J. S., Yue Q., Liu Y. Y., Jiao F., Kang Y. J. (2019). Two-dimensional copper nanosheets for electrochemical
reduction of carbon monoxide to acetate. Nat.
Catal..

[9] Haynes C. A., Gonzalez R. (2014). Rethinking biological activation of methane and conversion
to liquid fuels. Nat. Chem. Biol..

[10] Chen Z., Li P., Anderson R., Wang X., Zhang X., Robison L., Redfern L. R., Moribe S., Islamoglu T., Gomez-Gualdron D. A., Yildirim T., Stoddart J. F., Farha O. K. (2020). Balancing
volumetric and gravimetric uptake in highly porous materials for clean
energy. Science.

[11] Wang X., Xu A., Li F., Hung S. F., Nam D. H., Gabardo C. M., Wang Z., Xu Y., Ozden A., Rasouli A. S., Ip A. H., Sinton D., Sargent E. H. (2020). Efficient methane
electrosynthesis enabled by tuning local CO_2_ availability. J. Am. Chem. Soc..

[12] Kuhl K. P., Cave E. R., Abram D. N., Jaramillo T. F. (2012). New insights
into the electrochemical reduction of carbon dioxide on metallic copper
surfaces. Energy Environ. Sci..

[13] Kortlever R., Shen J., Schouten K. J., Calle-Vallejo F., Koper M. T. (2015). Catalysts and Reaction Pathways for
the Electrochemical
Reduction of Carbon Dioxide. J. Phys. Chem.
Lett..

[14] Iyengar P., Huang J., De Gregorio G. L., Gadiyar C., Buonsanti R. (2019). Size dependent
selectivity of Cu nano-octahedra catalysts for the electrochemical
reduction of CO_2_ to CH_4_. Chem. Commun..

[15] Rong W., Zou H., Zang W., Xi S., Wei S., Long B., Hu J., Ji Y., Duan L. (2021). Size-Dependent Activity and Selectivity
of Atomic-Level Copper Nanoclusters during CO/CO_2_ Electroreduction. Angew. Chem., Int. Ed..

[16] Fan Q., Zhang X., Ge X., Bai L., He D., Qu Y., Kong C., Bi J., Ding D., Cao Y., Duan X., Wang J., Yang J., Wu Y. (2021). Manipulating
Cu nanoparticle surface oxidation states tunes catalytic selectivity
toward CH_4_ or C_2+_ products in CO_2_ electroreduction. Adv. Energy Mater..

[17] Zhou X., Shan J., Chen L., Xia B. Y., Ling T., Duan J., Jiao Y., Zheng Y., Qiao S. Z. (2022). Stabilizing
Cu^2+^ ions by solid solutions to promote CO_2_ electroreduction
to methane. J. Am. Chem. Soc..

[18] Dai Y., Li H., Wang C., Xue W., Zhang M., Zhao D., Xue J., Li J., Luo L., Liu C., Li X., Cui P., Jiang Q., Zheng T., Gu S., Zhang Y., Xiao J., Xia C., Zeng J. (2023). Manipulating local
coordination of copper single atom catalyst enables efficient CO_2_-to-CH_4_ conversion. Nat.
Commun..

[19] Zou H., Zhao G., Dai H., Dong H., Luo W., Wang L., Lu Z., Luo Y., Zhang G., Duan L. (2023). Electronic Perturbation of Copper
Single-Atom CO_2_ Reduction
Catalysts in a Molecular Way. Angew. Chem.,
Int. Ed..

[20] Varandili S. B., Huang J. F., Oveisi E., De Gregorio G. L., Mensi M., Strach M., Vavra J., Gadiyar C., Bhowmik A., Buonsanti R. (2019). Synthesis of Cu/CeO_2‑x_ nanocrystalline heterodimers with interfacial active sites to promote
CO_2_ electroreduction. ACS Catal..

[21] Xiong L., Fu X., Zhang J., Liu S., Li S., Lu S., Wang D., Yue Q. (2025). Unraveling
the Mechanism of Cu/CeO_2_ Interface Modulation for CO_2_ Electroreduction
Into C_2+_/CH_4_. Adv. Funct.
Mater..

[22] Wei D.-Y., Zhang G., Wang H.-J., Zheng Q.-N., Tian J.-H., Zhang H., Li J.-F. (2024). Understanding
water-gas shift reaction
mechanisms at palladium-ceria interfaces using in situ SERS coupled
with online mass spectrometry. J. Mater. Chem.
A.

[23] Xia C., Wang X., He C., Qi R., Zhu D., Lu R., Li F. M., Chen Y., Chen S., You B., Yao T., Guo W., Song F., Wang Z., Xia B. Y. (2024). Highly
Selective Electrocatalytic CO_2_ Conversion to Tailored Products
through Precise Regulation of Hydrogenation and C-C Coupling. J. Am. Chem. Soc..

[24] Seo M., Chung T. D. (2019). Nanoconfinement effects in electrochemical reactions. Curr. Opin. Electroche..

[25] Wordsworth J., Benedetti T. M., Somerville S. V., Schuhmann W., Tilley R. D., Gooding J. J. (2022). The Influence of
Nanoconfinement
on Electrocatalysis. Angew. Chem., Int. Ed..

[26] Chang K., Jian X., Jeong H. M., Kwon Y., Lu Q., Cheng M.-J. (2020). Improving CO_2_ Electrochemical Reduction
to CO Using Space Confinement between Gold or Silver Nanoparticles. J. Phys. Chem. Lett..

[27] Ma M., Djanashvili K., Smith W. A. (2016). Controllable Hydrocarbon Formation
from the Electrochemical Reduction of CO_2_ over Cu Nanowire
Arrays. Angew. Chem., Int. Ed..

[28] Wordsworth J., Benedetti T. M., Alinezhad A., Tilley R. D., Edwards M. A., Schuhmann W., Gooding J. J. (2020). The importance of nanoscale confinement
to electrocatalytic performance. Chem. Sci..

[29] Hall A. S., Yoon Y., Wuttig A., Surendranath Y. (2015). Mesostructure-Induced
Selectivity in CO_2_ Reduction Catalysis. J. Am. Chem. Soc..

[30] Yang P. P., Zhang X. L., Gao F. Y., Zheng Y. R., Niu Z. Z., Yu X., Liu R., Wu Z. Z., Qin S., Chi L. P., Duan Y., Ma T., Zheng X. S., Zhu J. F., Wang H. J., Gao M. R., Yu S. H. (2020). Protecting Copper
Oxidation State via Intermediate Confinement for Selective CO_2_ Electroreduction to C_2+_ Fuels. J. Am. Chem. Soc..

[31] Ma D., Lu H., Zhou Y., Jiang S., Wang D., Yue Q. (2024). A Novel Molten
Salt Mediated Synthesis of Mesoporous Metal Oxides with High Crystallization. ACS Cent. Sci..

[32] Roget F., Favotto C., Rogez J. (2013). Study of the KNO_3_-LiNO_3_ and KNO_3_-NaNO_3_-LiNO_3_ eutectics
as phase change materials for thermal storage in a low-temperature
solar power plant. Sol. Energy.

[33] Cao T., You R., Zhang X., Chen S., Li D., Zhang Z., Huang W. (2018). An in situ
DRIFTS mechanistic study of CeO_2_-catalyzed
acetylene semihydrogenation reaction. Phys.
Chem. Chem. Phys..

[34] Gunathunge C. M., Ovalle V. J., Li Y., Janik M. J., Waegele M. M. (2018). Existence
of an electrochemically inert CO population on Cu electrodes in alkaline
pH. ACS Catal..

[35] Zhang L., Feng J., Wang R., Wu L., Song X., Jin X., Tan X., Jia S., Ma X., Jing L., Zhu Q., Kang X., Zhang J., Sun X., Han B. (2025). Switching
CO-to-Acetate Electroreduction on Cu Atomic Ensembles. J. Am. Chem. Soc..

[36] Liu Q., Jiang Q., Li L., Yang W. (2024). Spontaneous Reconstruction
of Copper Active Sites during the Alkaline CORR: Degradation and Recovery
of the Performance. J. Am. Chem. Soc..

[37] Huang F., Chen X., Sun H., Zeng Q., Ma J., Wei D., Zhu J., Chen Z., Liang T., Yin X., Liu X., Xu J., He H. (2025). Atmosphere Induces Tunable Oxygen
Vacancies to Stabilize Single-Atom Copper in Ceria for Robust Electrocatalytic
CO_2_ Reduction to CH_4_. Angew. Chem., Int. Ed..

[38] Pu Y., Luo Y., Wei X., Sun J., Li L., Zou W., Dong L. (2019). Synergistic effects of Cu_2_O-decorated CeO_2_ on
photocatalytic CO_2_ reduction: Surface lewis acid/base and
oxygen defect. Appl. Catal., B.

[39] Fan L., Geng Q., Ma L., Wang C., Li J.-X., Zhu W., Shao R., Li W., Feng X., Yamauchi Y., Li C., Jiang L. (2023). Evoking C_2+_ production from electrochemical
CO_2_ reduction by the steric confinement effect of ordered
porous Cu_2_O. Chem. Sci..

[40] Cheng T., Xiao H., Goddard W. A. (2015). Free-energy
barriers and reaction
mechanisms for the electrochemical reduction of CO on the Cu(100)
surface, including multiple layers of explicit solvent at pH 0. J. Phys. Chem. Lett..

[41] Li J., Chang X., Zhang H., Malkani A. S., Cheng M. J., Xu B., Lu Q. (2021). Electrokinetic and in situ spectroscopic investigations
of CO electrochemical reduction on copper. Nat.
Commun..

[42] Xiong L., Fu X. (2025). Recent advances and
challenges in electrochemical CO_2_ reduction
to CH_4_. Chin. J. Catal..

[43] Valencia D., Martinez-Hernandez E., Aburto J. (2020). Effect of confinement space on adsorption
energy and electronic structure of molecule-metal pairs. Struct. Chem..

[44] Pan F., Duan X., Fang L., Li H., Xu Z., Wang Y., Wang T., Li T., Duan Z., Chen K. J. (2024). Long-Range Confinement-Driven Enrichment of Surface
Oxygen-Relevant Species Promotes C-C Electrocoupling in CO_2_ Reduction. Adv. Energy Mater..

[45] Kim Y., Yun G. T., Kim M., Jamal A., Gereige I., Ager J. W., Jung W. B., Jung H. T. (2024). Effect of Feature
Shape and Dimension of a Confinement Geometry on Selectivity of Electrocatalytic
CO_2_ Reduction. Angew. Chem., Int.
Ed..

[46] Wang J., Qin Y., Jin S., Yang Y., Zhu J., Li X., Lv X., Fu J., Hong Z., Su Y., Wu H. B. (2023). Customizing
CO_2_ Electroreduction by Pulse-Induced Anion Enrichment. J. Am. Chem. Soc..

[47] Wang D., Liu Z.-P., Yang W.-M. (2017). Proton-promoted electron transfer
in photocatalysis: key step for photocatalytic hydrogen evolution
on metal/titania composites. ACS Catal..

[48] Wang D., Liu Z.-P., Yang W.-M. (2018). Revealing
the size effect of platinum
cocatalyst for photocatalytic hydrogen evolution on TiO_2_ support: a DFT study. ACS Catal..

[49] Akhade S. A., Luo W., Nie X., Asthagiri A., Janik M. J. (2016). Theoretical insight
on reactivity trends in CO_2_ electroreduction across transition
metals. Catal. Sci. Technol..

[50] Meng Y., Xu Z., Shen Z., Xia Q., Cao Y., Wang Y., Li X. (2022). Understanding the water
molecule effect in metal-free B-based electrocatalysts
for electrochemical CO_2_ reduction. J. Mater. Chem. A.

[51] Cheng Q., Wei H., Wang J., Wang Z.-Q., Gong X.-Q., Wang D. (2024). Clarifying
the Direct Generation of •OH Radicals in Photocatalytic O_2_ Reduction: Theoretical Prediction Combined with Experimental
Validation. J. Phys.Chem. Lett..

[52] Wang D., Sheng T., Chen J., Wang H.-F., Hu P. (2018). Identifying
the key obstacle in photocatalytic oxygen evolution on rutile TiO_2_. Nat. Catal..

[53] Li F., Chen J.-F., Gong X.-Q., Hu P., Wang D. (2022). Subtle structure
matters: the vicinity of surface Ti_5c_ cations alters the
photooxidation behaviors of anatase and rutile TiO_2_ under
aqueous environments. ACS Catal..

[54] Bae J. H., Han J.-H., Chung T. D. (2012). Electrochemistry
at nanoporous interfaces:
new opportunity for electrocatalysis. Phys.
Chem. Chem. Phys..

