# Supplementary material for: Unveiling the Nanoconfinement Effect in CO2 Electroreduction to CH4 over Mesoporous Cu-CeO2 Nanospheres
Source: ACS Cent Sci. 2025 Aug 22;11(10):1902–10. doi: 10.1021/acscentsci.5c01035 (PMC12550617; doi:10.1021/acscentsci.5c01035)
Supplement: Supplementary file 1 [file oc5c01035_si_001.pdf]

Supporting information

Unveiling the Nanoconfinement Effect in CO_2 Electroreduction to CH_4 over Mesoporous Cu-CeO_2 Nanospheres

Lei Xiong,[a] Xianbiao Fu,[b] Wenpu Fan,[c] Jun Zhang,[d] Zixuan Zheng,[e] Shaojie Lu,[a] Dong Wang,[c] Mingze Hao,[a] and Qin Yue[a]*

[a] Institute of Fundamental and Frontier Sciences, University of Electronic Science and Technology of China, Chengdu, 610054, China.

[b] Department of Materials Science and Engineering, National University of Singapore, Singapore 117576, Singapore.

[c] State Key Laboratory of Green Chemical Engineering and Industrial Catalysis, Centre for Computational Chemistry and Research Institute of Industrial Catalysis, School of Chemistry and Molecular Engineering, East China University of Science and Technology, 130 Meilong Road, Shanghai 200237, China

[d] State Key Laboratory of Industrial Vent Gas Reuse, Southwest Institute of Chemical Co., Ltd., Chengdu 610225, China

[e] College of Chemistry, Sichuan University, Chengdu 610040, China

Corresponding author. E-mail: qinyue@uestc.edu.cn

Experimental section

Chemicals

$Ce(SO_4)_2{\cdot}4H_2O$, $Zr(SO_4)_2{\cdot}4H_2O$, KNO_3, $LiNO_3$ and $Cu(NO_3)_2{\cdot}3H_2O$ were all purchased from Aladdin. Pluronic F127 was purchased from Sigma-Aldrich. The carbon paper with gas diffusion layer (GDL) was purchased from Toray Industries.

Synthesis of Cu/mCeO_2-x catalysts

In a typical synthesis of Cu/mCeO_2-3.0, 1 mmol $Ce(SO_4)_2{\cdot}4H_2O$ and 10 mmol nitrate (molar ratio KNO_3:$LiNO_3$=0.57:0.43) were mixed and ground in a mortar for 5 min. Then F127 (400 mg, ~24 wt% of the total amount) was added and ground for another 5 minutes. The mixture was transferred to the crucible and calcined in the muffle furnace. The temperature was firstly increased from room temperature to 160 °C with heating rate of 2 $°C{\cdot}min^{-1}$ and kept for 3 h. Then it was further raised to 400 °C with the same heating rate and kept for another 3 h. After cooling down to the room temperature, the salt was removed by washing and centrifugation with deionized water for more than four times. The remaining solid was dried in the oven at 70 °C to get the mesoporous CeO_2 microspheres (mCeO_2). The related mCeO_2 was served as carriers to prepare the Cu/mCeO_2-x catalysts (theoretical Cu loading content of 3 wt%) via the impregnation method. Typically, 100 mg of the mCeO_2 was dispersed in 5 mL $Cu(NO_3)_2$ solution (9.4 mM) with ultrasonication for 30 min. Then the suspension was constantly stirred at 90 °C to evaporate the water. The solid was dried at 70 °C and then calcined under Ar atmosphere at 350 °C for 2 h (heating rate of 2 $°C{\cdot}min^{-1}$) to obtain the catalyst labeled as Cu-mCeO_2-3.0. The catalysts Cu-mCeO_2-1.3, Cu-mCeO_2-4.3 and Cu-mCeO_2-5.5 are prepared via the same procedure besides the amount of F127 in the synthesis of mCeO_2 were 14 wt% (200 mg), 32 wt% (600 mg) and 39 wt% (800 mg), respectively.

Synthesis of Cu/mZrO_2-x catalysts

The synthesis of the three mesoporous Cu/ZrO_2-*x* (*x*=1.2, 2.8 and 4.0 nm) catalysts is similar with that of Cu/mCeO_2-*x* (*x*=1.3, 3.0 and 4.3 nm) with the same F127 amount

(200, 400, 600 mg) except the precursor $Ce(SO_4)_2·4H_2O$ is replaced by $Zr(SO_4)_2·4H_2O$.

Material Characterizations

X-ray diffraction patterns (XRD) were characterized on a D/Max-2500 spectrometer (Rigaku, USA) by using Cu Kα rays (λ = 0.15406 nm) radiation in the 2θ range of 10-90°. Feld emission scanning electron microscope (FE-SEM) was conducted on MIRA3 at 10 kV (TESCAN, The Czech Republic). Transmission electron microscopy (TEM) images were obtained on Talos F200X (Thermo Fisher Scientific, USA) at 200 kV equipped with energy dispersive spectroscopy (EDS). The X-ray photoelectron spectra (XPS) was collected on Escalab Xi+ (Thermo Fisher Scientific, USA). The actual Cu content was determined by the Inductively Coupled Plasma Optical Emission Spectrometer (ICP-OES) in a PerkinElmer 8300 spectrometer. Prior to measurements, all samples were firstly digested in concentrated HNO_3 and diluted in DI water with specific concentrations. The LabRam HR Evolution laser confocal micro-Raman spectrometer was employed to record the Raman spectra with laser wavelength of 532 nm. The N_2 adsorption-desorption isotherm was characterized on the TriStar 3000 sorption analyzer (Micromeritics, USA) for Cu-mCeO_2-1.3 and Quadrosorb evo sorption analyzer (Quantachrome, USA) for another three catalysts at 77 K. Prior to the experiment, the sample was degassed at 300 °C for 3 h under vacuum. The Brunauer-Emmett-Teller (BET) and Density Functional Theory (DFT) methods were used to obtain the specific surface area and pore size distribution, respectively.

The in situ diffuse reflectance infrared Fourier transform spectroscopy (DRIFTS) was used to characterize the physical state of Cu for the CO adsorption over Cu-mCeO_2-*x* catalyst, which was performed on a Thermo Nicolet 6700 Spectrometer (Thermo Fisher, USA) equipped with high-temperature reaction cell and MCT detector. The IR spectra were obtained with a resolution of 64 scans and 4 cm^{-1} in absorbance units. 100 mg of catalyst were firstly heated up to 300 °C and kept for 30 min in flowing He gas (40 mL·min^{-1}), and then switched to 5 vol% H_2/He treatment for another 30 min. The sample was cooled down to 25 °C in flowing He (40 mL·min^{-1}) and the spectra were taken for a background. Afterwards, the gas was changed to CO/He (2 vol%, 30

mL·min^{-1}) and retained for 30 min until saturation coverage. At last, the gas was switched to He (40 mL/min) and retained for 5 min before the spectra were collected.

CO_2 temperature programmed desorption (CO_2-TPD) was performed on TP-5076 TPD/TPR dynamic adsorber (Xianquan, China) in a quartz tubular microreactor equipped with a mass spectrometry (MS) detector. 50 mg of the sample was firstly activated in flowing He at 300 ºC for 30 min and then cooled down to 25 ºC. The adsorption was carried out at 25 ºC for 30 min under constant stream of pure CO_2 (20 mL·min^{-1}). Subsequently, CO_2 desorption was started in flowing He (20 mL·min^{-1}) under temperature from 25 ºC to 500 ºC with heating rate of 10 ºC·min^{-1}. The CO_2 signal (m/z=44) was continuously recorded online in a mass spectrometer (EXQ, Hiden).

In-situ ATR-SEIRAS experiments

The in-situ ATR-SEIRAS of the catalysts was performed on an INVENIO R FT-IR spectrometer (Bruker, German) equipped with a MCT detector. Typically, Pt sheet and Ag/AgCl electrode were used as the counter electrode and reference electrode, respectively. For the working electrode, 30 uL of catalyst ink (5 mg·mL^{-1}) was dropped onto a semi-cylindrical Si crystal and then vacuum dried. Then a stainless-steel wire was glued on the catalyst edge to lead out. 10 mL of 0.5 M $KHCO_3$ solution was used as the electrolyte. Pure CO_2 gas (20 mL·min^{-1}) was continuously pumped into the electrolyte during the experiments. The working electrode was firstly pre-reduced at -1.0 V (vs. RHE) for 20 min to make the working electrode at a steady state. Then the ATR-SEIRAS tests were performed between -0.3 and -1.0 V (vs. RHE) with pre-recorded background spectra. All spectra were recorded by absorbance that was log(R/R_0). The spectral resolution and scan rate was 4 cm^{-1} and 3 mv·s^{-1}, respectively. The scan time was 0.5 min.

Preparation of gas diffusion electrodes

12 mg catalyst powder was dispersed in 4 mL isopropanol solution containing 20 μL Nafion ionomer solution (5 wt% H_2O) with sonication for 30 min to form uniform

ink. Then, the catalyst ink was drop-cast onto the carbon paper equipped with gas diffusion layer (GDL) (Toray, Japan) to achieve a catalyst loading of 1 mg·m^{-2} and serve as the gas diffusion electrode (GDE).

Electrochemical measurement

Electrochemical surface area (ECSA) of Cu-mCeO_{2-x} were measured by Pb underpotential deposition (UPD) method. The electrolyte of 0.1 M $HClO_4$ and 1 mM $PbCl_2$ aqueous solution (pH 1.4) was purged with Ar gas at least 0.5 h. After CO_2RR test, the electrodes were transferred and cyclic voltammetry was measured from -0.05 V to -0.65 V vs Ag/AgCl reference electrode in a scan rate of 50 mV/s. The area of a Pb monolayer stripping peak around -0.3 V vs Ag/AgCl was integrated to calculate the transferred charge. The ECSAs of the Cu-based electrodes were estimated with the conversion factor of 310 μC/cm^2 assuming Pb adatoms on Cu and 2e$^-$ Pb oxidation.[1, 2] The electrochemical impedance spectroscopy (EIS) was measured at an applied potential of -0.8 V (vs RHE) with an amplitude of 5 mV and frequency range from 0.01 Hz to 100 kHz.

CO_2 electrolyzing experiments were implemented on a customized three-chamber flow cell with effective area of 1 cm^2 (1 cm ×1 cm) with three electrode system. The prepared GDE, Ni foam and Ag/AgCl (saturated KCl solution) were served as cathode working electrode, counter electrode and reference electrode, respectively. A piece of proton exchange membrane (Nafion-117) was used to separate cathode and anode compartments. The electrolyte was 0.5 M $KHCO_3$ aqueous solution (99.99%, Macklin Inc.), which was saturated by pure CO_2 for 30 min before test. Using a syringe pump, the cathode and anode chambers were constantly fed with the electrolyte with flow rate of 1.0 mL·min^{-1}. 20 sccm of CO_2 feedstock (99.99%) was supplied to cathode gas chamber controlled by a mass flow-meter. The CO_2 electrolysis was conducted by chronoamperometry method. All recorded potentials were transformed to reverse hydrogen electrode (RHE) according to equation $E_{RHE} = E_{Ag/AgCl} + E^0_{Ag/AgCl} + 0.059 \times pH$ (V). The measured bias prior to electrolysis was iR-corrected with 90%, while the resistance between the cathode and the reference electrode was determined by the

current-interruption method.

Using a gas chromatography spectrometry (GC-2014C, Shimadzu) with online system, the gas products were analyzed, in which H_2 and CO were quantified by thermal conductivity detector (TCD) and flame ionization detector (FID1), while CH_4 and C_2H_4 were detected on FID2. The faradic efficiency of gas products was determined through the equation:

$$\mathrm{FE}(\%) = \frac{Q_x}{Q_{total}} \times 100\% = \frac{n_x \times C_x \times vt \times F}{I \times t \times V_M} \times 100\% = \frac{n_x \times C_x \times v \times F}{I \times V_M} \times 100\%$$

The liquid products were then analyzed using a Bruker AVIII (600 MHz) nuclear magnetic resonance hydrogen spectrometer (^{1}HMR). Specifically, after 20 min of electrolysis, 500 μl of cathodic electrolyte containing the liquid products was mixed with 100 μl of D_2O containing 20 ppm (v/v) dimethyl sulfoxide (≥99.9%, Alfa Aesar) as internal standard. Then the one-dimensional ^{1}HMR spectra of the liquid products under different current density were measured. The product amount was quantified by the peak area of the corresponding species. The Faradaic efficiency of the liquid product y was then determined by the following equation:

$$\mathrm{FE}(\%) = \frac{Q_y}{Q_{total}} \times 100\% = \frac{n_y \times C_y \times v_L t \times F}{I \times t} \times 100\% = \frac{n_y \times C_y \times v_L \times F}{I} \times 100\%$$

n_x, n_y: electrons transferred for reduction to product x, y;

C_x, C_y: volume fraction of product x, or y quantified by GC or ^{1}HMR;

v: molar gas flow velocity of outlet, mol·s^{-1};

v_L: Cathode liquid flow rate, L·s^{-1}.

F: Faradic constant, 96500 C·mol^{-1};

I: average current during electrolysis, A;

V_M: gas molar volume, 22.4 L·mol^{-1}.

Computational methods and Models

All density functional theory (DFT) calculations were performed using the VASP package[3, 4] with the spin-polarization being considered. The DFT functional was utilized at the Perdew-Burke-Ernzerhof level. The projector-augmented wave (PAW) method with an energy cutoff of 450 eV was employed to describe the interaction

between atomic cores and electrons, with the Ce (5s, 5p, 6s, 5d, 4f), Cu (4s, 3d), O (2s, 2p), C (2s,2p) and H (1s) shells being treated as valence electrons.[5] Throughout the calculations, we applied the on-site Coulomb interaction correction with an effective U value of 4.5 eV for the Ce 4f orbitals to better describe the localized electron states, consistent with the previous reports.[5-7] The transition states (TSs) of surface reactions were located using a constrained minimization method[6,7] and verified by the vibrational analysis. The DFT-D3 scheme was applied throughout the calculations to take the van der Waals (vdW) interaction into account. All the calculations were converged until the Hellman–Feynman forces on each ion were less than 0.05 eV/Å. Adopting the approach, the lattice parameter of CeO_2 unit cell was calculated to be a=b=c=5.423 Å, well consistent with the experimental value of a=b=c=5.411 Å. The adsorption energy (E_{ad}) was calculated according to $E_{ad} = E_{tot} - E_{sur} - E_X$, where E_{tot} represents for the total energy of the interacting adsorbate plus catalyst model, E_{sur} for the average energy of confined models, and E_X for the adsorbate energy referencing to CO, H_2O, and H_2 molecules in the gas phase. Obviously, a negative value corresponds to an exothermic adsorption process, and the more negative the E_{ad} is, the stronger the adsorbate binds on the surface.

For the model construction, the CeO_2(111) surface, was constructed as a four-Ce-layer periodic slab composed of 144 atoms, on which single Cu atoms were uniformly dispersed at the most stable sites with a mass fraction of 3 wt% (Figure 5a (1)). All four Ce-layers were allowed to fully relax during the structural optimization, using a 1×1×1 K-point mesh for the Brillouin-zone integration. To simulate the confinement effect in cylindrical pores of experimental materials, we adopted a representative model engaging one-dimensional confined space in z axis, which has been proven effective in capturing intermolecular interactions in confined space.[8-10] Thus, different confinement degrees among virous pore size in experiments were readily simulated by varying the vacuum distance between two adjacent slabs. It should be noted that, for molecules/species in small cylindrical pores, the confined space equivalent to the corresponding diameter (i.e. at the centerline) actually introduces the less confinement effect, whereas the off-centerline positions (confined space shorter than the diameter)

give stronger confinement interactions. Therefore, in addition to the minimum pore size of 13 Å in experimental materials, we also considered even smaller size of 6 Å and 9 Å as well as larger ones (18 Å and 24 Å) to account for different degrees of confinement effects in those porous materials.

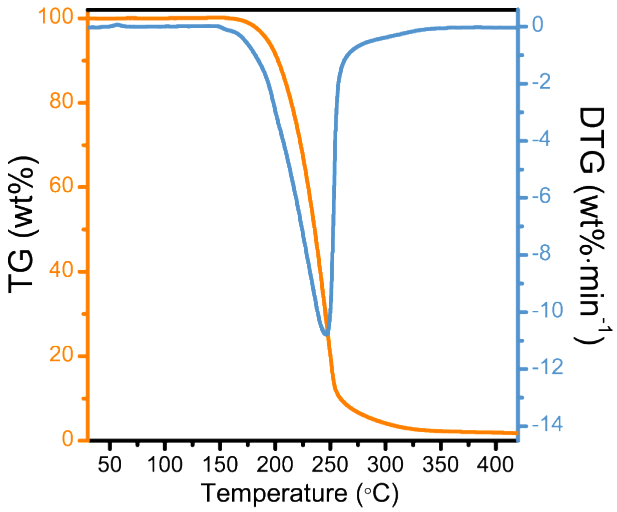

Figure S1. The thermogravimetric curves of F127.

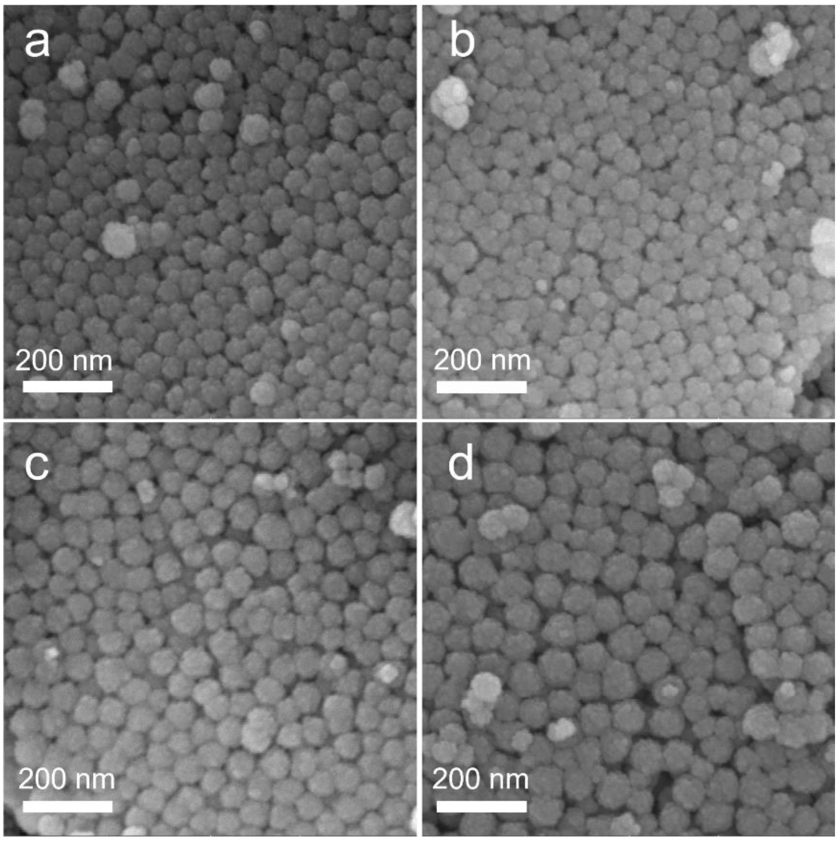

Figure S2. The SEM images of the Cu-CeO$_2$-x catalysts. (a) Cu-mCeO$_2$-1.3, (b) Cu-mCeO$_2$-3.0, (c) Cu-mCeO$_2$-4.3, and (d) Cu-mCeO$_2$-5.5.

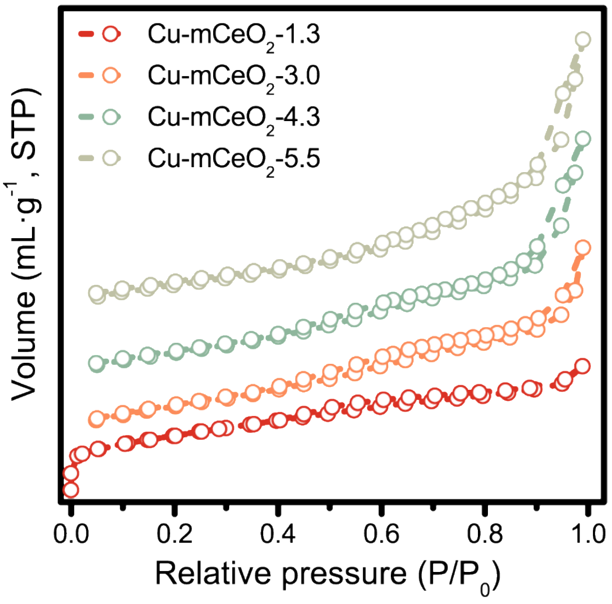

Figure S3. The N_2 adsorption-desorption isotherm of Cu-CeO_2-*x* catalysts.

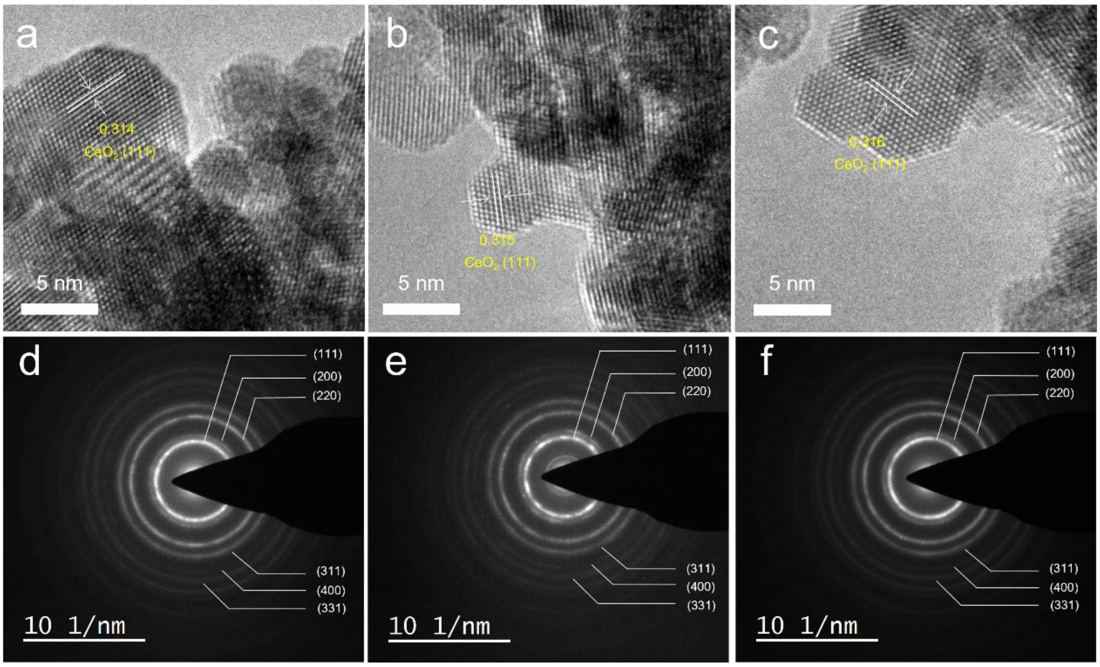

Figure S4. The HRTEM images (a-c) and relative SAED (d-f) for Cu-mCeO_2-1.3 (a, d), Cu-mCeO_2-4.3 (b, e) and Cu-mCeO_2-5.5 (c, f).

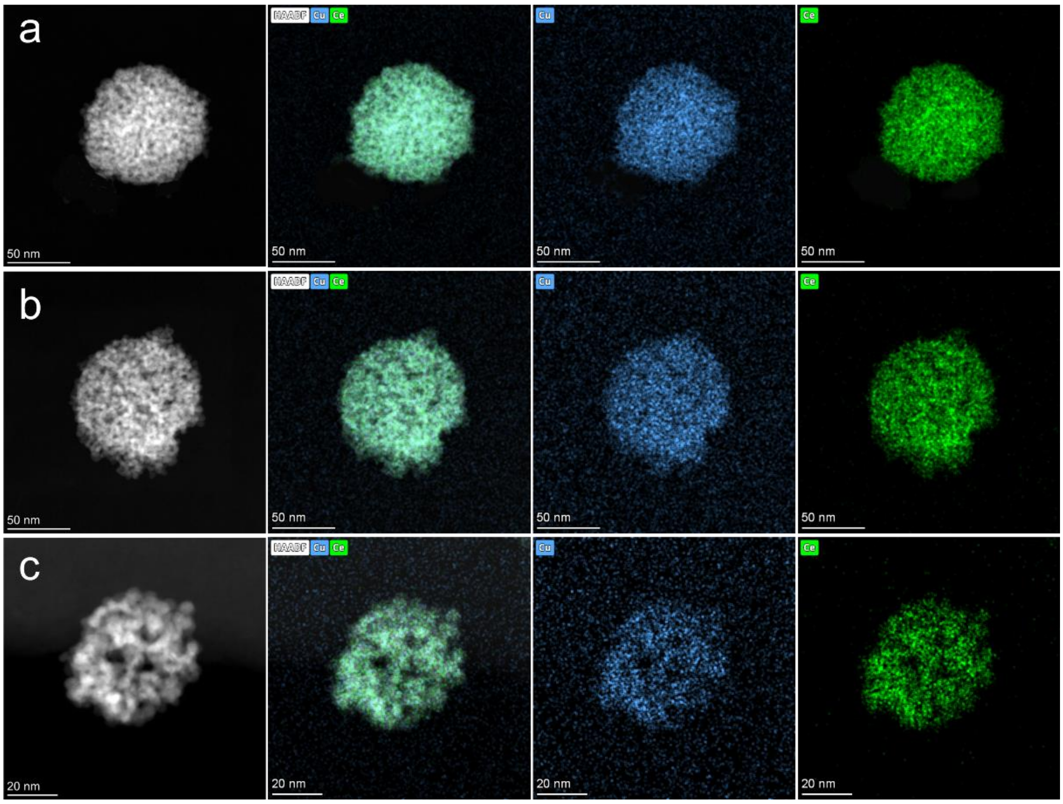

Figure S5. The EDS mappings of (a) Cu-mCeO_2-1.3, (b) Cu-mCeO_2-4.3, and (c) Cu-mCeO_2-5.5.

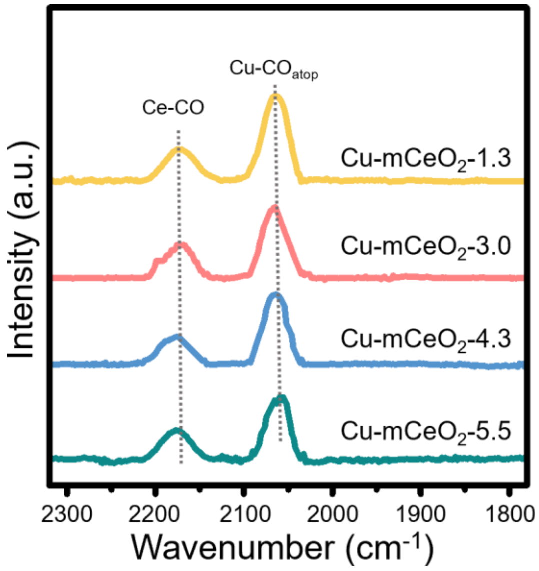

Figure S6. The CO Drifts characterizations of the four Cu-mCeO$_2$-*x* catalysts.

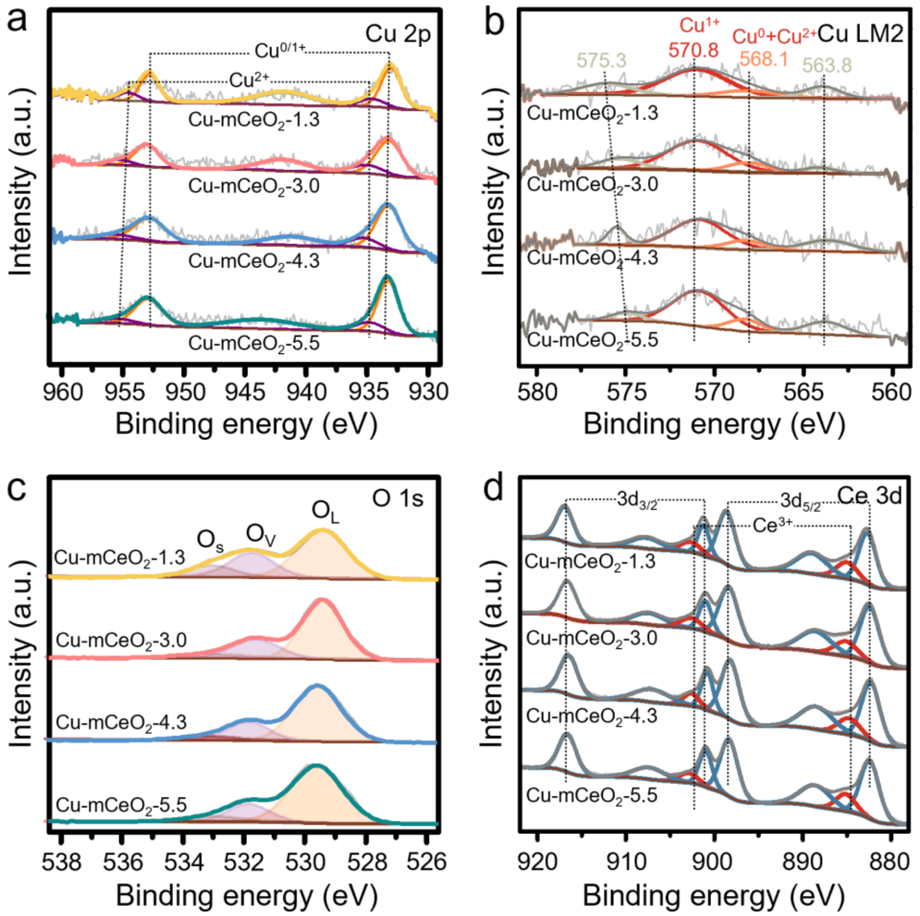

Figure S7. The Cu 2p (a), Cu LM2 (b), O1s (c) and Ce 3d (d) XPS spectra of Cu-$mCeO_2$-*x* catalysts.

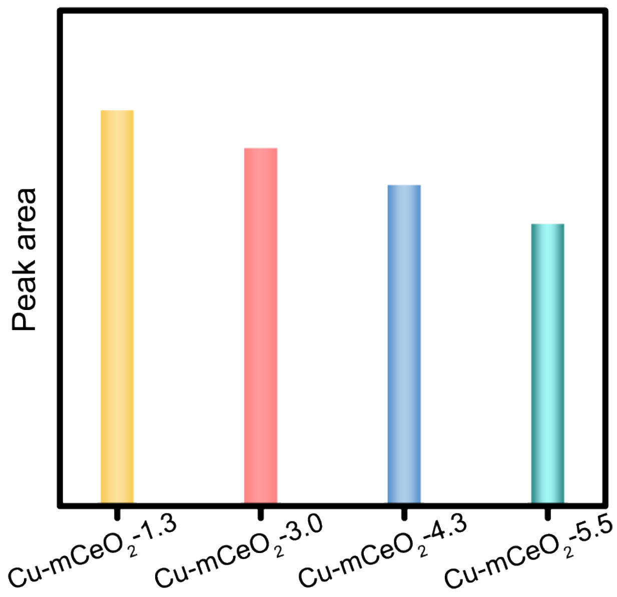

Figure S8. The CO_2 desorption peak area of the Cu-mCeO_2-*x* catalysts.

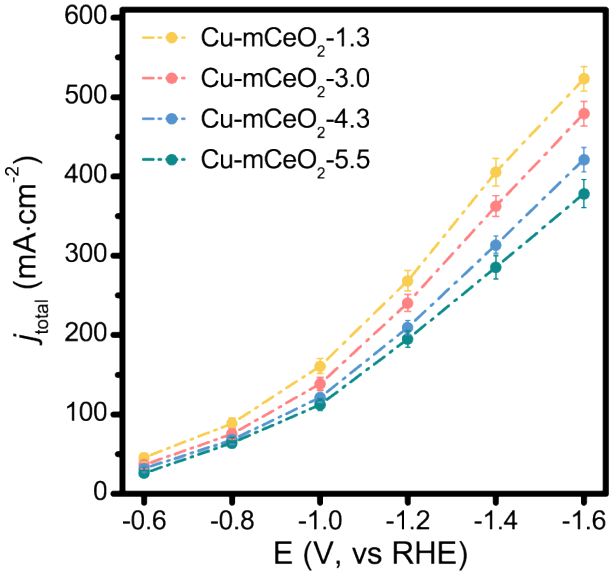

Figure S9. The total current density (j_{total}) of various catalysts.

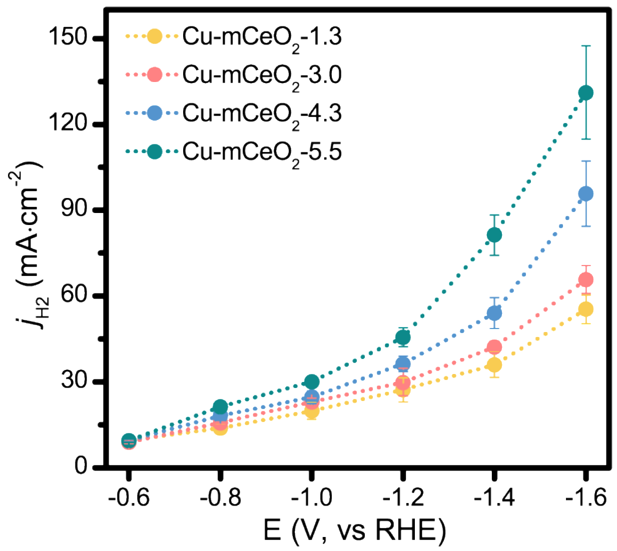

Figure S10. The H_2 partial current density (j_{H2}) of the Cu-mCeO_2-x catalysts.

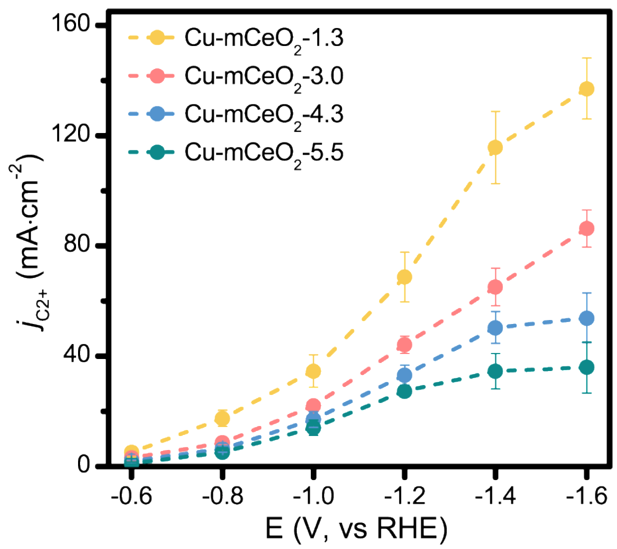

Figure S11. The partial current density (j_{C2+}) of C_{2+} products (C_2H_4, C_2H_5OH, CH_3COOH) for the Cu-mCeO_2-x catalysts.

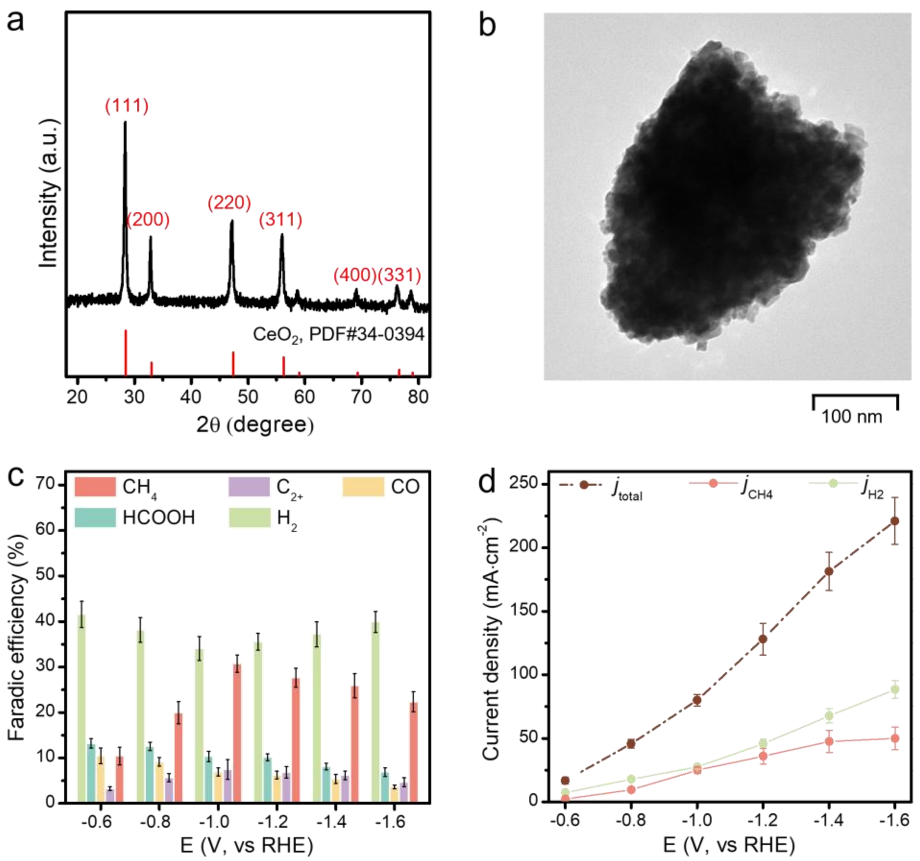

Figure S12. The characterizations of (a) XRD patterns, (b) TEM image and CO_2RR performance of (c) products FE, (d) current density for Cu-cCeO_2.

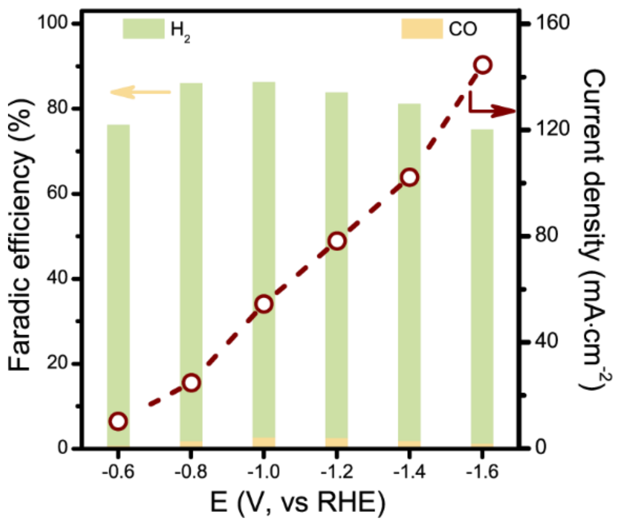

Figure S13. The CO_2RR products faradic efficiency and total current density of pristine CeO_2.

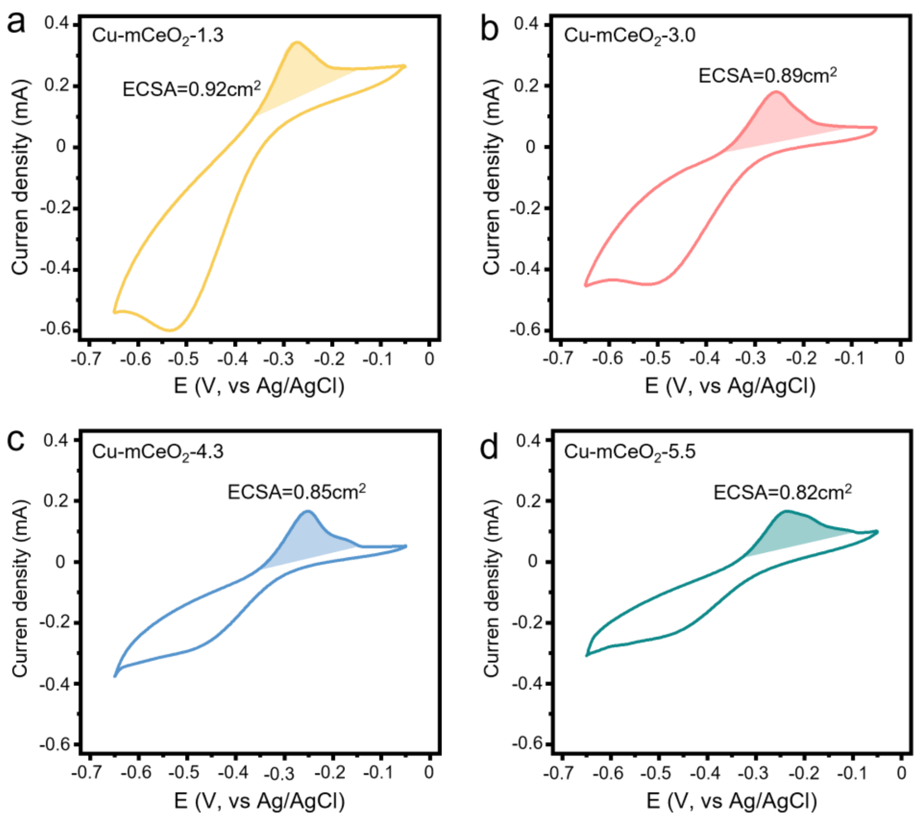

Figure S14. The CV curves of electrocatalysts in Ar-saturated 0.01M $HClO_4$ + 1 mM $PbCl_2$ solution at a scan rate of 50 mV·s^{-1} for Cu-mCeO_2-1.3 (a), Cu-mCeO_2-3.0 (b), Cu-mCeO_2-4.3 (c), and Cu-mCeO_2-5.5 (d).

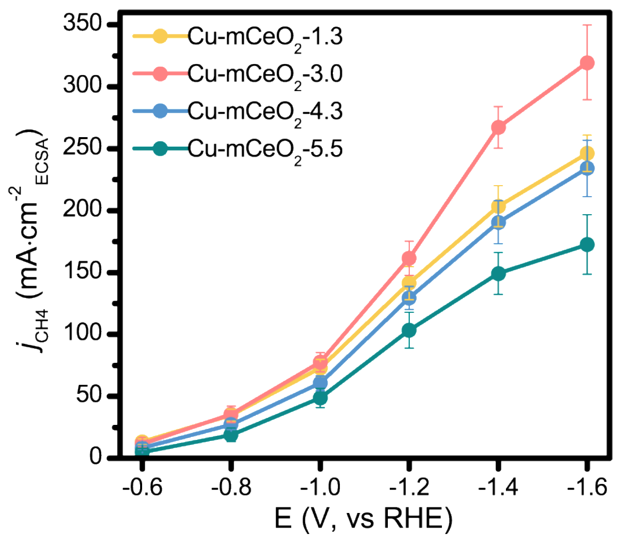

Figure S15. The ECSA normalized j_{CH4} for Cu-mCeO$_2$-x catalysts.

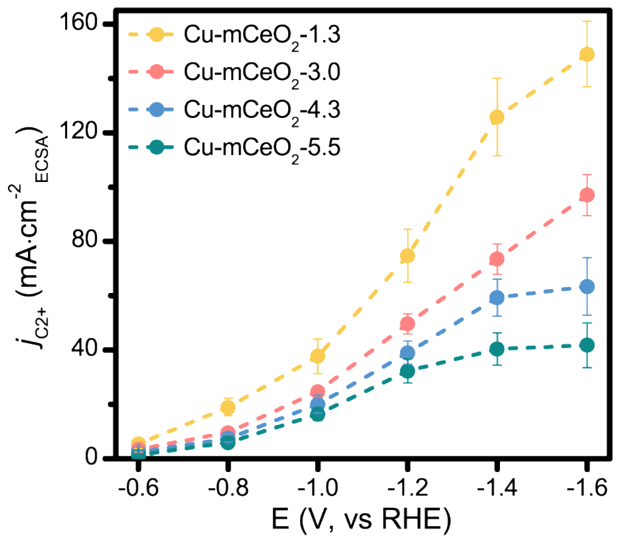

Figure S16. The ECSA normalized j_{C2+} for Cu-mCeO_2-x catalysts.

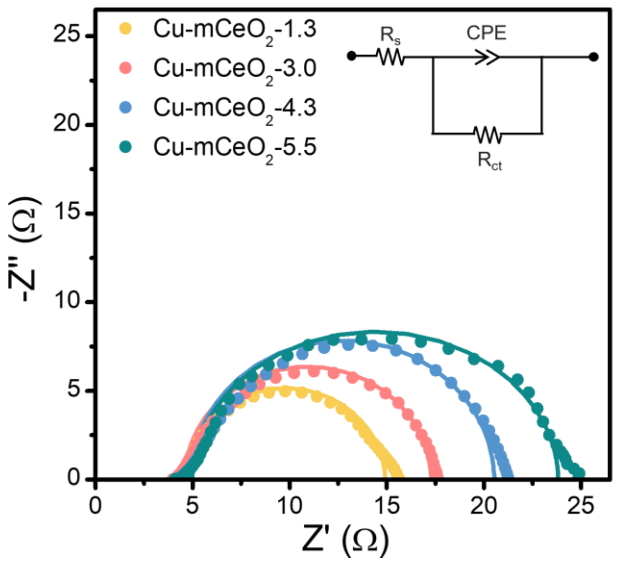

Figure S17. The EIS measurements of the Cu-mCeO_2-*x* catalysts.

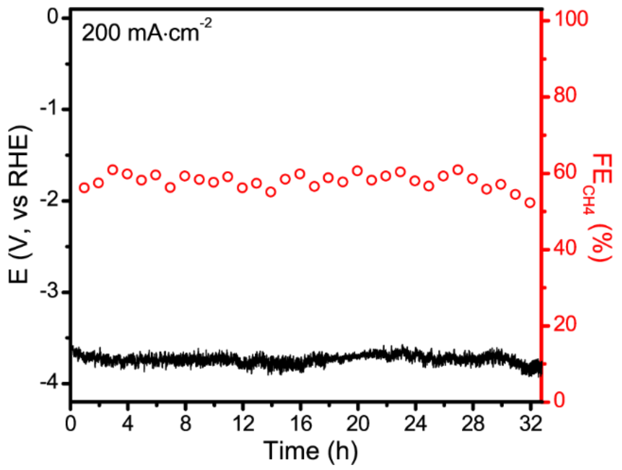

Figure S18. The long-term stability test of Cu-mCeO_2-3.0 under current density of 200 mA cm^{-2} in a MEA device.

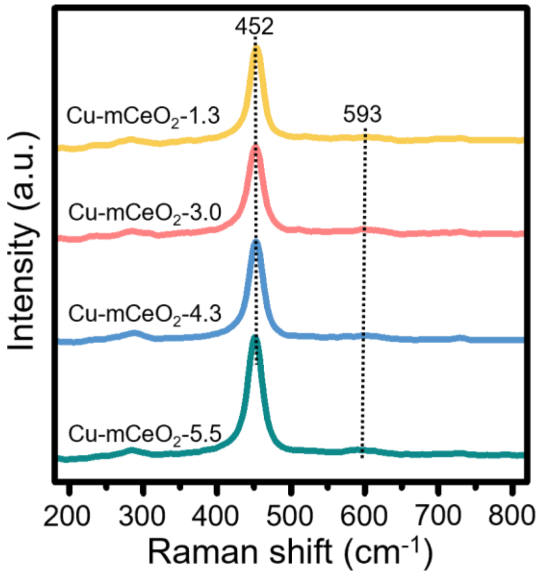

Figure S19. The Raman spectra of the catalysts after CO_2RR.

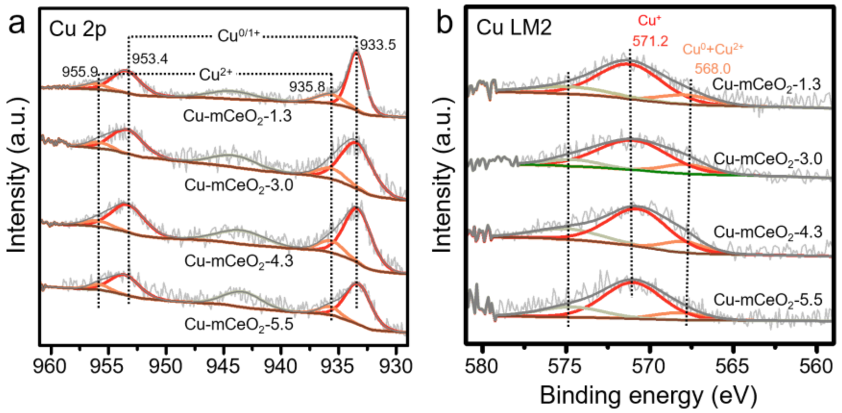

Figure S20. The Cu 2p XPS (a) and Cu LM2 XPS (b) spectra of the four catalysts after CO_2RR.

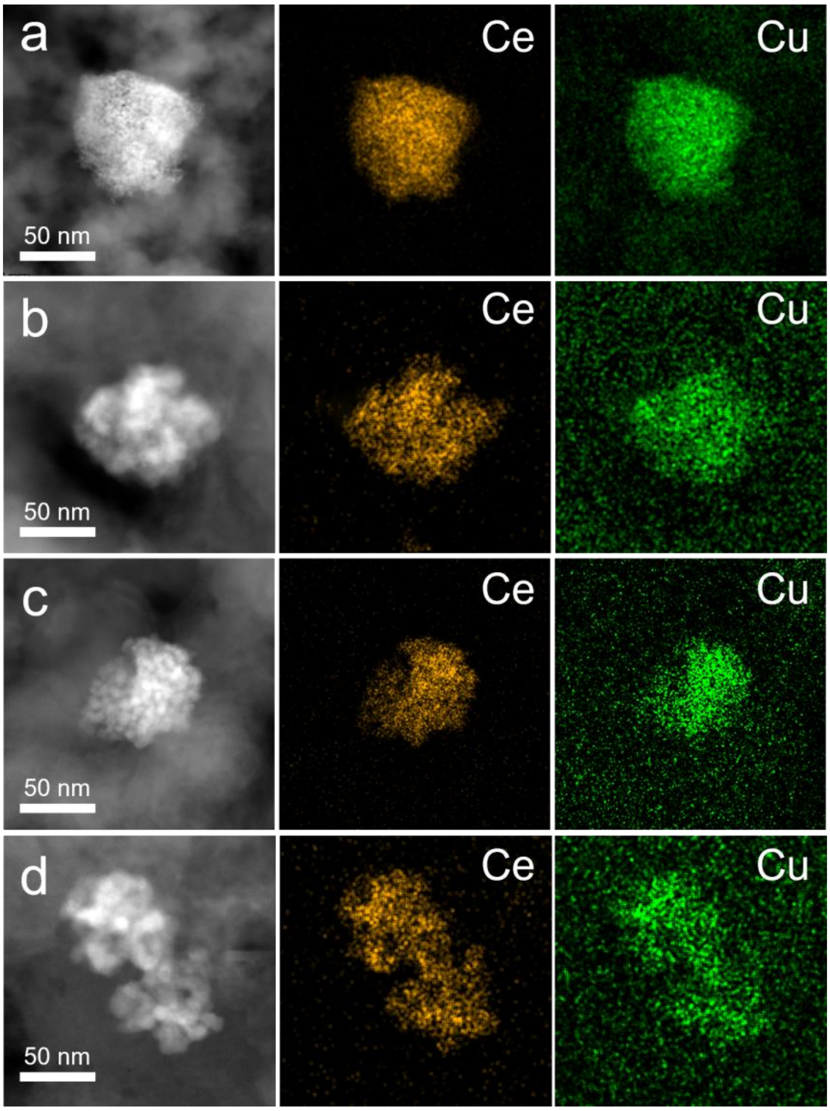

Figure S21. The EDS mappings of (a) Cu-mCeO_2-1.3, (b) Cu-mCeO_2-3.0, (c) Cu-mCeO_2-4.3, and (d) Cu-mCeO_2-5.5.

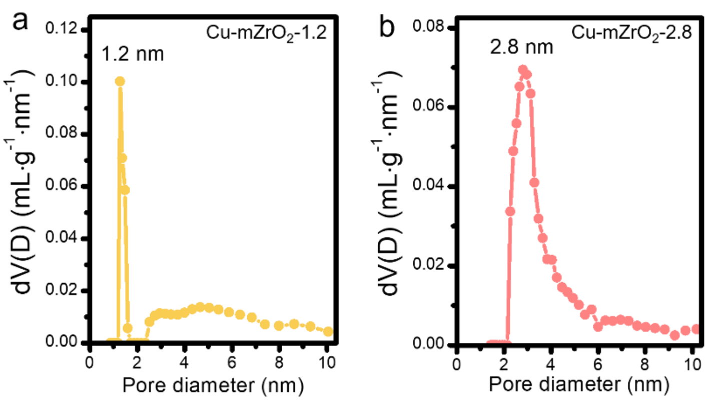

Figure S22. The pore size distribution of Cu-ZrO_2-*x* catalysts derived from the N_2 adsorption-desorption characterizations.

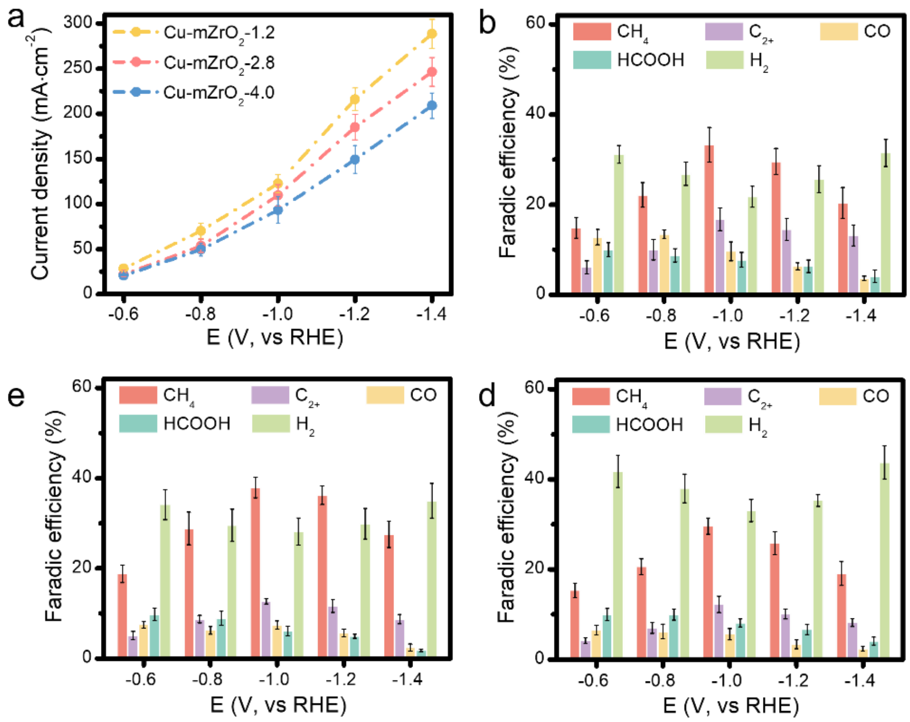

Figure S23. The CO_2RR performance of the Cu-mZrO_2-x catalysts. (a) The total current density (j_{total}) of the catalysts. The FEs for (b) Cu-mCeO_2-1.2, (c) Cu-mCeO_2-2.8 and (d) Cu-mCeO_2-4.0.

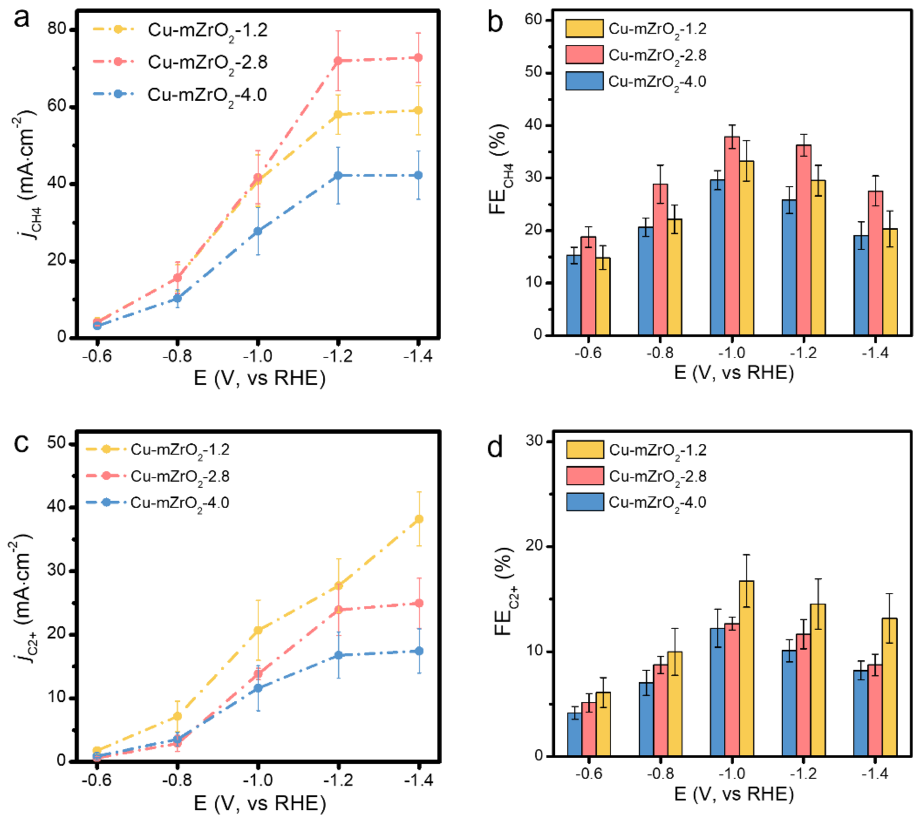

Figure S24. The relative product selectivity of CH_4 (a), (b) and C_{2+} (c), (d) for the three Cu-mZrO_2-*x* catalysts.

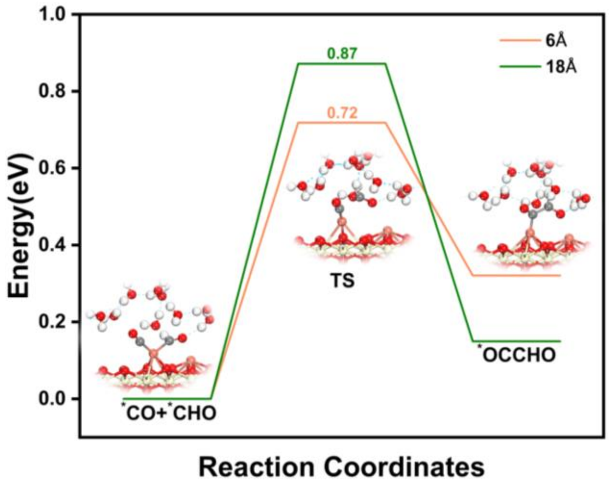

Figure S25. Reaction barriers for the coupling of *CO and *CHO to form C_{2+} in aqueous environments within two distinct confined spaces. This was done by introducing local water molecules (the first solvation shell) around the reaction center explicitly, forming hydrogen-bonding network with the reactant and thereby mimicking the aqueous environment, which is also a typical approach for simulating water surroundings.[8-12]

Table S1. The textural results of Cu-mCeO_2-*x* catalysts.

	Surface area ($m^2 \cdot g^{-1}$)	Pore volume ($mL \cdot g^{-1}$)
**Cu-mCeO_2-1.3**	165	0.16
**Cu-mCeO_2-3.0**	151	0.28
**Cu-mCeO_2-4.3**	140	0.34
**Cu-mCeO_2-5.5**	126	0.38

Table S2. The surface atomic percentage from XPS results.

	O_v (%)	Cu^{2+} (%)	Cu^{+} (%)
**Cu/mCeO$_2$-1.3**	26.8	19.6	80.1
**Cu/mCeO$_2$-3.0**	24.7	19.2	80.4
**Cu/mCeO$_2$-4.3**	21.1	18.9	80.6
**Cu/mCeO$_2$-5.5**	19.2	18.3	81.4

Table S3. The simulation results of the EIS curves for the catalysts.

	R_s (Ω)	R_{ct} (Ω)
**Cu-mCeO_2-1.3**	4.5	10.4
**Cu-mCeO_2-3.0**	4.6	12.7
**Cu-mCeO_2-4.3**	4.8	15.6
**Cu-mCeO_2-5.5**	4.8	16.6

Table S4. The surface Cu^{x+} percentage from XPS results after CO_2RR tests.

	Cu^{2+} (%)	Cu^{+} (%)	Cu content (wt%)
**Cu-mCeO$_2$-1.3**	17.6	81.7	2.96
**Cu-mCeO$_2$-3.0**	16.6	82.8	3.06
**Cu-mCeO$_2$-4.3**	15.5	82.9	3.02
**Cu-mCeO$_2$-5.5**	15.1	84.2	2.91

References

(1) Jung, H.; Lee, S. Y.; Lee, C. W.; Cho, M. K.; Won, D. H.; Kim, C.; Oh, H. S.; Min, B. K.; Hwang, Y. J. Electrochemical Fragmentation of Cu_2O Nanoparticles Enhancing Selective C-C Coupling from CO_2 Reduction Reaction. *J. Am. Chem. Soc.* **2019**, *141*, 4624-4633.

(2) Chen, H.-Q.; Zhao, W.; Wang, L.; Chen, Z.; Ye, W.; Zang, J.; Wang, T.; Sun, L.; Yang, W. Origin of Metal–Support Interactions for Selective Electrochemical CO_2 Reduction into C_1 and C_{2+} Products. *ACS Catal.* **2024**, *14*, 11794-11802.

(3) G. Kresse, J. F. Efficiency of ab-initio total energy calculations for metals and semiconductors using a plane-wave basis set. *Comp. Mater. Sci.* **1996**, *6*, 15-50.

(4) Kresse, G.; Hafner, J. Ab initio molecular-dynamics simulation of the liquid-metal-amorphous-semiconductor transition in germanium. *Phys. Rev. B* **1994**, *49*, 14251-14269.

(5) Zheng, Z.-Y.; Wang, D.; Zhang, Y.; Yang, F.; Gong, X.-Q. Structures and reactivities of the CeO_2/Pt(111) reverse catalyst: A DFT+U study. *Chinese J. Catal.* **2020**, *41*, 1360-1368.

(6) Zhou, H.; Wang, D.; Gong, X.-Q. Clarifying the impacts of surface hydroxyls on CO oxidation on CeO_2(100) surfaces: a DFT+U study. *Phys. Chem. Chem. Phys.* **2020**, *22*, 7738-7746.

(7) Zhou, C.-Y.; Wang, D.; Gong, X.-Q. A DFT+U revisit of reconstructed CeO_2(100) surfaces: structures, thermostabilities and reactivities. *Phys. Chem. Chem. Phys.* **2019**, *21*, 19987-19994.

(8) Wang, J.; Qin, Y.; Jin, S.; Yang, Y.; Zhu, J.; Li, X.; Lv, X.; Fu, J.; Hong, Z.; Su, Y.; Wu, H. B. Customizing CO_2 Electroreduction by Pulse-Induced Anion Enrichment. *J. Am. Chem. Soc.* **2023**, *145*, 26213-26221.

(9) Wang, D.; Liu, Z.-P.; Yang, W.-M. Proton-promoted electron transfer in photocatalysis: key step for photocatalytic hydrogen evolution on metal/titania composites. *ACS Catal.* **2017**, *7*, 2744-2752.

(10) Wang, D.; Liu, Z.-P.; Yang, W.-M. Revealing the size effect of platinum cocatalyst for photocatalytic hydrogen evolution on TiO_2 support: a DFT study. *ACS Catal.* **2018**,

8, 7270-7278.

(11) Akhade, S. A.; Luo, W.; Nie, X.; Asthagiri, A.; Janik, M. J. Theoretical insight on reactivity trends in CO_2 electroreduction across transition metals. *Catal. Sci. Technol.* **2016**, *6*, 1042-1053.

(12) Meng, Y.; Xu, Z.; Shen, Z.; Xia, Q.; Cao, Y.; Wang, Y.; Li, X. Understanding the water molecule effect in metal-free B-based electrocatalysts for electrochemical CO_2 reduction. *J. Mater. Chem. A* **2022**, *10*, 6508-6522.
